# Lactylation of SLC26A3 in the acidic tumor microenvironment promotes malignant progression of colorectal carcinoma

**DOI:** 10.1038/s41419-026-08422-9

**Published:** 2026-01-30

**Authors:** Chong Chen, Du Cai, Xuanhui Liu, Yifan Zheng, Xinxin Huang, Dongwen Chen, Jiawei Cai, Yiran Bie, Zhengran Zhou, Chuling Hu, Zhengyu Wei, Kuntai Cai, Ting Li, Shuzhen Luo, Dongbing Liu, Kui Wu, Zerong Cai, Feng Gao, Xiaojian Wu, Peishan Hu

**Affiliations:** 1 Department of General Surgery (Colorectal Surgery), The Sixth Affiliated Hospital, Sun Yat-sen University, Guangzhou, PR China; 2 Guangdong Provincial Key Laboratory of Colorectal and Pelvic Floor Diseases, The Sixth Affiliated Hospital, Sun Yat-Sen University, Guangzhou, PR China; 3 Biomedical Innovation Center, The Sixth Affiliated Hospital, Sun Yat-sen University, Guangzhou, PR China; 4 Precision Medicine Center, Affiliated Hospital of Guangdong Medical University, Zhanjiang, 524001 Guangdong PR China; 5 Department of Gastroenterology and Urology, The Affiliated Cancer Hospital of Xiangya School of Medicine, Central South University/Hunan Cancer Hospital, Changsha, 410013 PR China; 6 Institute of Intelligent Medical Research (IIMR), BGI Genomics, Shenzhen, PR China; 7 Guangdong Provincial Key Laboratory of Human Disease Genomics, BGI Research, Shenzhen, PR China

**Keywords:** Gastrointestinal cancer, Cancer

## Abstract

The acidic tumor microenvironment provides the energy that drives the development of malignant tumors. High concentrations of lactic acid and H^+^ are key features of the acidic tumor microenvironment, and lactylation has gradually been shown to play a significant role in tumor progression. The expression of solute carrier family 26 member 3 (SLC26A3) is closely related to the occurrence and development of colorectal cancer (CRC), but the specific molecular mechanisms remain unclear. We demonstrated that alterations in the acidic microenvironment and overexpression of SLC26A3 significantly inhibited CRC occurrence and progression in vivo. Our study indicates that SLC26A3 undergoes lactylation in the acidic tumor microenvironment, which decreases SLC26A3 stability and expression. SLC26A3 interacts with the RNA-binding proteins Hu antigen R (HuR) and CUG-binding protein 1 (CUGBP1). When SLC26A3 expression is reduced, its ability to bind to HuR/CUGBP1 is weakened. As a result, HuR and CUGBP1 more readily interact with a subset of oncogenic mRNAs, regulating their stability and influencing their expression, ultimately promoting malignant tumor progression. These findings highlight the role of SLC26A3 as a potential suppressor of CRC recurrence, drug resistance, and metastasis, providing new insights for improving the clinical treatment and prognosis of CRC.

## Introduction

Colorectal cancer (CRC) is one of the most common malignant tumors, ranking third in incidence and second in mortality among primary tumors globally in 2022 [1]. Despite the availability of various treatment options for CRC, including surgery, chemotherapy, and radiotherapy, the prognosis for CRC remains poor, with a 5-year survival rate of only 13.1% for patients with metastatic CRC [2]. Tumor recurrence, metastasis, and drug resistance are key factors contributing to poor outcomes and mortality in CRC patients. Cancer stem cells play crucial roles in tumorigenesis, drug resistance, and metastasis [3], and the tumor microenvironment is a critical determinant in determining the phenotype of cancer stem cells [4, 5]. The tumor microenvironment helps maintain the key characteristics of cancer stem cells, preserves their phenotypic plasticity, protects them from immune system attacks, and enhances their metastatic potential [6]. Therefore, elucidating the molecular mechanisms by which the tumor microenvironment regulates tumor stem cell phenotypes and identifying key targets for CRC recurrence, drug resistance, and metastasis will provide new insights for improving CRC treatment strategies and patient outcomes.

Acidity is a key physicochemical characteristic of the tumor microenvironment that influences various stages of tumorigenesis [7–10]. Recent studies have shown that the acidic tumor microenvironment in gastrointestinal tumors induces tumor stem cell phenotypes and metabolic reprogramming, thereby promoting malignant tumor progression [11]. High lactic acid and H^+^ concentrations are major features of the acidic tumor microenvironment, and lactylation, a novel post-translational modification discovered in recent years, involves the modification of proteins by lactic acid and plays a significant role in the regulation of metabolism and gene expression [12–14]. Although studies have revealed that the potential importance of lactylation in tumor progression, our understanding of its mechanisms and functions is still in its early stages. Therefore, we aimed to investigate the impact of lactylation in the acidic tumor microenvironment on malignant tumor progression.

SLC26A3 is an ion channel protein that is primarily expressed in colon cells and is responsible for the transport of Cl^−^ and HCO_3_^−^ [15]. SLC26A3 is considered a suppressor of CRC, as its expression is significantly reduced in CRC and CRC cell lines compared to normal controls; thus, its encoded protein was named downregulated in adenoma [16, 17]. The downregulation of SLC26A3 is associated with the malignant progression of CRC [18], but its molecular mechanisms of action and whether SLC26A3 regulates tumor stem cell phenotypes remain unclear.

In this study, we found that the acidic tumor microenvironment reduced the expression of SLC26A3 by promoting its lactylation, thereby inhibiting the interaction between SLC26A3 and HuR/CUGBP1. As a result, HuR/CUGBP1 bound more to the malignant mRNA subset, regulating its stability and promoting tumor cell stemness, invasion, migration, epithelial-mesenchymal transition (EMT), and drug resistance. Therefore, this study revealed a novel mechanism by which the SLC26A3-HuR/CUGBP1 signaling axis regulated the progression of CRC.

## Results

### SLC26A3 expression is negatively correlated with acidic micro-environment-associated CRC malignancy

**Fig. 1:**
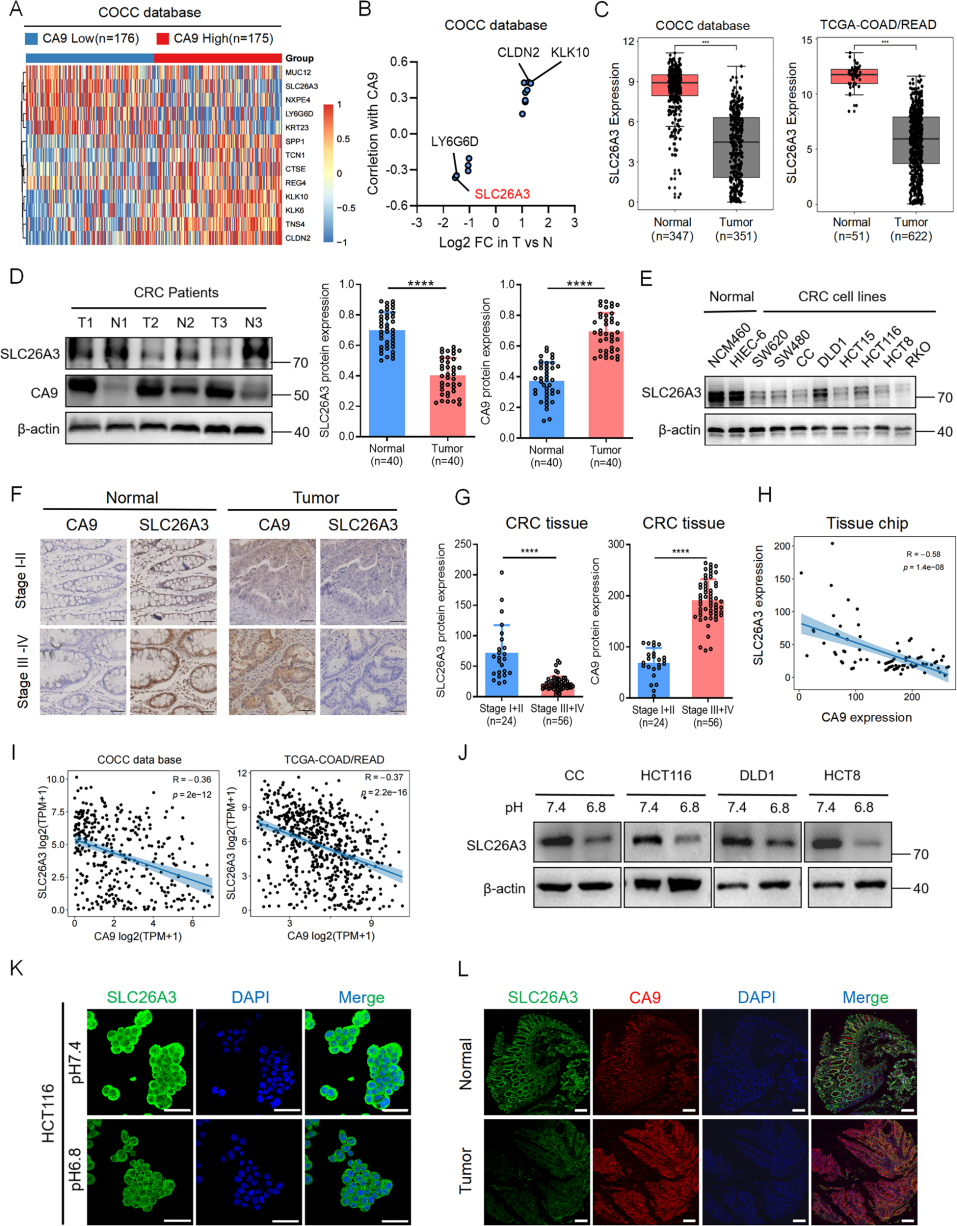
SLC26A3 expression is negatively correlated with acidic microenvironment-associated CRC malignancy. **A** Genes significantly related to the expression of the acid marker CA9 were screened out from the COCC database. **B** The expression level of SLC26A3 is significantly correlated with CA9, and the expression level is significantly different between tumor and normal tissues. **C** Expression of SLC26A3 in tumor and normal tissues in COCC and TCGA databases. Wilcox rank sum test. **D** Immunoblotting of SLC26A3 in normal and CRC tissue from representative patients (*n* = 40) in our hospital (SYSUSH). Quantified immunoblotting results using ImageJ. Student’s *t* test. **E** Immunoblotting of SLC26A3 in normal cell lines and CRC cell lines. Tissue chip immunohistochemistry (**F**) and H-Score (**G**) of SLC26A3 and CA9 in normal and tumor tissues from CRC patients in different stages. Scale bars: 50 μm. Student’s *t* test. **H** Correlation analysis of SLC26A3 and CA9 expression in tissue chip. Pearson correlation analysis. **I** Correlation analysis of SLC26A3 and CA9 expression in COCC and TCGA databases. Pearson correlation analysis. **J** Immunoblotting of SLC26A3 in different CRC cell lines under pH 7.4 and 6.8. **K** Immunofluorescence of SLC26A3 in HCT116 cells under pH 7.4 and 6.8. Scale bars: 50 μm. **L** Multiplex immunofluorescence staining of SLC26A3 and CA9 in tissue chips of CRC patients. Scale bars: 200 μm. *P < 0.05, **P < 0.01, and ***P < 0.001, Data are representative of three independent experiments.

To investigate the key molecules regulating the malignant phenotype of CRC in an acidic microenvironment, we analyzed data from the clinical omics study of colorectal cancer in China (COCC) database (part of the ICGC-ARGO project undertaken by the Sixth Affiliated Hospital of Sun Yat-Sen University, which involved 1001 patients who underwent radical surgery for CRC at our hospital). Based on the expression of the acidic environment marker carbonic anhydrase IX (CA9), 351 CRC tissue samples were divided into high and low CA9 expression groups. Genes that were significantly differentially expressed between the CA9 high and low-expression groups were screened, and the correlations between their expression and CA9 expression were analyzed (log_2_FC > 1, Fig. 1A). Among them, SLC26A3 exhibited significant differential expression between the high and low CA9 expression groups and had the strongest negative correlation with CA9 (Fig. 1B and Supplementary Fig. S1A). Analysis of paired normal and tumor tissues from the COCC and The Cancer Genome Atlas (TCGA) databases revealed that SLC26A3 was significantly down-regulated in CRC tissues (Fig. 1C). Moreover, SLC26A3 was also found to be expressed at low levels in most cancer types (Supplementary Fig. S1D). We further validated the low expression of SLC26A3 and its negative correlation with CA9 expression in tumor tissues from CRC patients in our hospital (Fig. 1D). Additionally, SLC26A3 was found to be down-regulated in CRC cell lines compared with normal intestinal epithelial cell lines (Fig. 1E and Supplementary Fig. S1B). Immunohistochemical staining of tissue microarrays from postoperative samples from patients with CRC (*n* = 80) at our hospital revealed that SLC26A3 expression was lower in stage III-IV patients than in stage I-II patients, whereas CA9 expression was higher in stage III-IV patients (Fig. 1F, G). The expression of these two proteins was negatively correlated (Fig. 1H). Analysis of the COCC and TCGA databases also revealed a negative correlation between SLC26A3 and CA9 expression (Fig. 1I). When CRC cells were cultured in pH 6.8 medium [11], SLC26A3 expression levels decreased after acid treatment (Fig. 1J and Supplementary Fig. S1C). Cell immunofluorescence experiments revealed that SLC26A3 was expressed at low levels in acidic environments (Fig. 1K), and SLC26A3 expression in tumor or normal tissues was negatively correlated with CA9 expression (Supplementary Fig. S1F). Multiplex immunofluorescence staining of the tissue chip also revealed that SLC26A3 expression was low in the tumor and was negatively correlated with CA9 expression (Fig. 1L and Supplementary Fig. S1E). In summary, low SLC26A3 expression is associated with malignant tumor status, suggesting that SLC26A3 may be a tumor suppressor gene, but the specific mechanism requires further clarification.

### Single-cell analysis reveals low SLC26A3 expression in the acidic tumor microenvironment

**Fig. 2:**
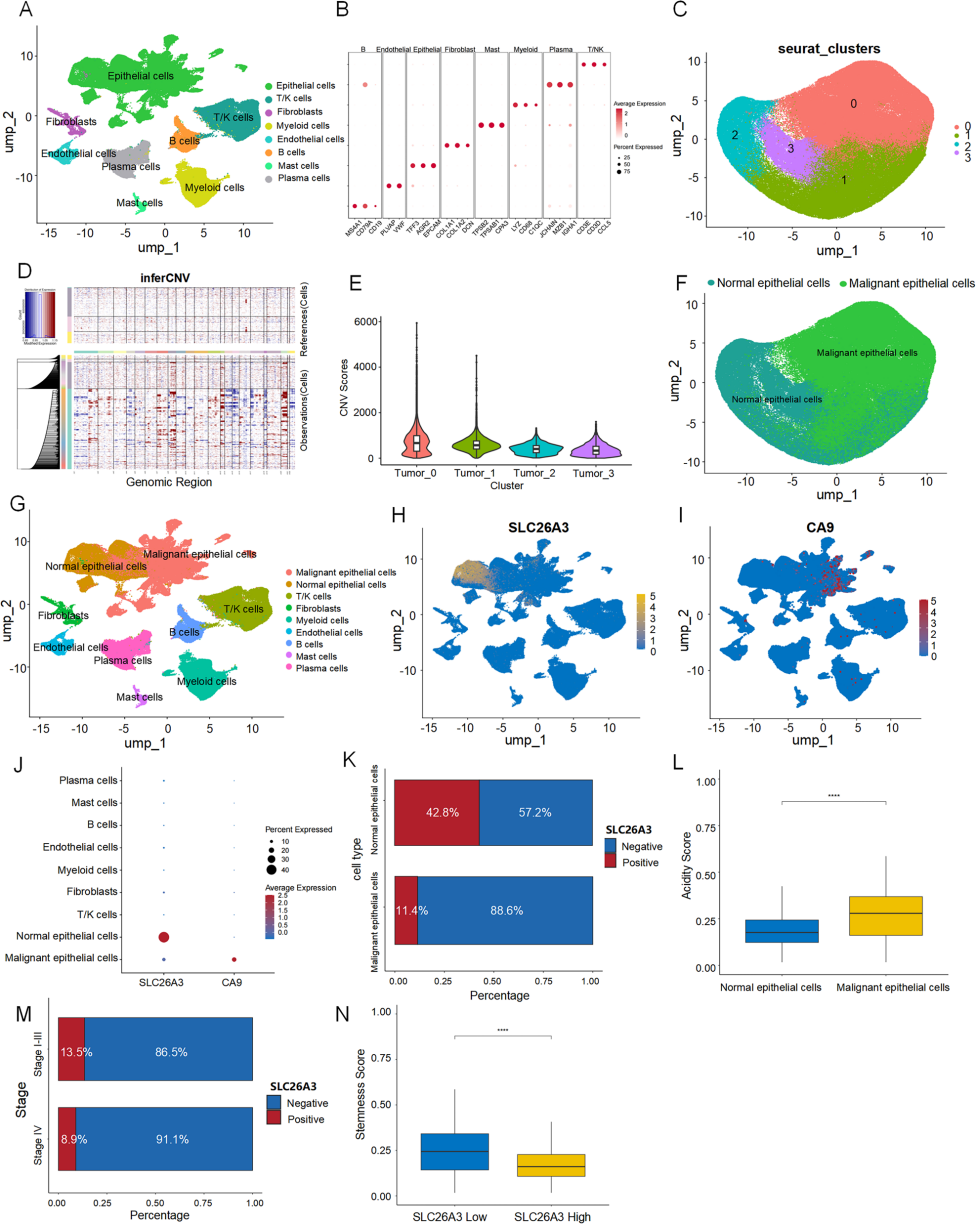
Single-cell analysis reveals low SLC26A3 expression in the acidic tumor microenvironment. **A** UMAP plot showing major cell types in the GSE178341 dataset. **B** Heatmap displaying the top 5 marker genes for each major cell type in the GSE178341 dataset. **C** UMAP plot illustrating the clustering of epithelial cells into four distinct subpopulations. **D** Heatmap depicting copy number variation (CNV) patterns across chromosomal regions for each epithelial subcluster. **E** Violin plot showing CNV scores of epithelial subclusters. **F** UMAP plot visualizing the distribution of normal epithelial cells and malignant epithelial cells. **G** UMAP plot annotating nine cell types identified in the GSE178341 dataset. **H** UMAP plot displaying the expression distribution of the SLC26A3 gene. **I** UMAP plot showing the expression distribution of the CA9 gene. **J** Dot plot comparing the expression of SLC26A3 and CA9 across different cell types. **K** Stacked bar chart representing the proportion of SLC26A3 expression in normal vs. malignant epithelial cells. **L** Box plot comparing the acidity scores of normal and malignant epithelial cells. Wilcox rank sum test. **M** Stacked bar chart demonstrating the proportion of SLC26A3 expression in epithelial cells across tumor stages (I-III vs. IV). **N** Box plot illustrating stemness scores in epithelial cells with high vs. low SLC26A3 expression. Wilcox rank sum test. *P < 0.05, **P < 0.01, and ***P < 0.001.

To investigate the expression profile of SLC26A3 and acidic microenvironment characteristics in colorectal tumor cells at single-cell resolution, we analyzed transcriptional profiles of 371,223 cells from 62 colorectal tumors and adjacent normal tissues in the public database (GSE178341). Based on classical marker gene expression, these cell clusters were annotated into eight major lineages: epithelial cells, T/NK cells, B cells, fibroblasts, myeloid cells, endothelial cells, mast cells, and plasma cells (Fig. 2A, B). Epithelial cells were subsequently extracted for re-clustering and dimensionality reduction, yielding four distinct subclusters (Fig. 2C). To identify malignant epithelial cells in CRC, we performed inferCNV analysis (Fig. 2D) and calculated copy number variation (CNV) scores for each epithelial subcluster (Fig. 2E), categorizing them into normal and malignant epithelial populations (Fig. 2F, G). Analysis of SLC26A3 expression across subpopulations revealed significantly lower levels in malignant epithelial cells. In contrast, the acidic marker CA9 was highly expressed in malignant epithelial cells (Fig. 2H–J). These findings indicate that SLC26A3 is downregulated in tumor tissues and the acidic microenvironment (Fig. 2K, L), consistent with our previous conclusions.

Further analysis of stage IV tumor patients demonstrated even lower SLC26A3 expression in epithelial cells compared to stages I-III (Fig. 2M). Malignant epithelial cells with low SLC26A3 expression exhibited elevated stemness scores (Fig. 2N). These results suggest that reduced SLC26A3 expression may enhance tumor cell stemness and metastatic potential, a hypothesis to be explored in subsequent studies.

### Lactylation of SLC26A3 in acidic environment reduces its stability and expression

**Fig. 3:**
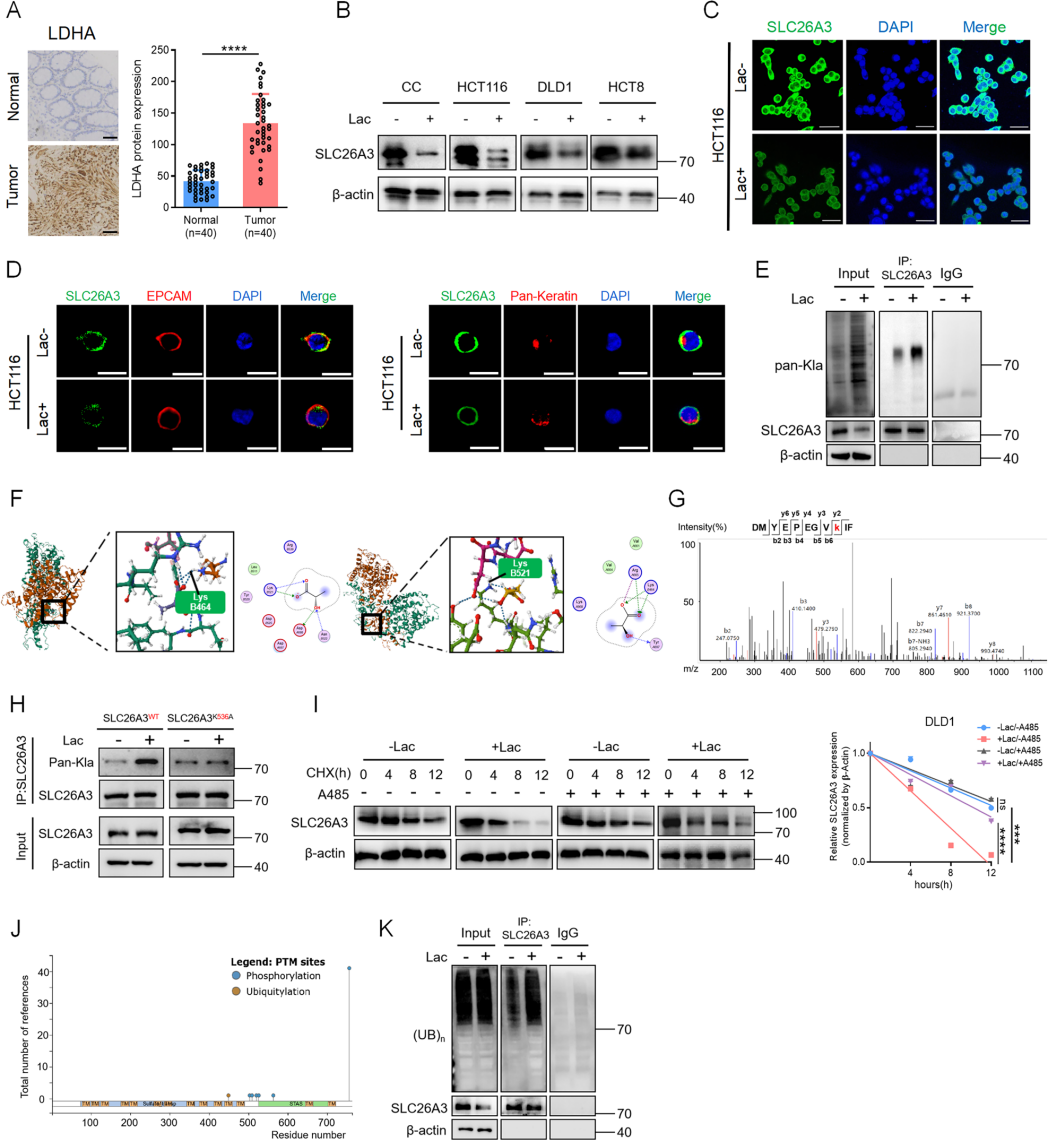
Lactylation of SLC26A3 in acidic environment reduces its stability and expression. **A** Tissue chip immunohistochemistry and H-Score of LDHA in normal and tumor tissues from CRC patients. Scale bars: 50 μm. Student’s *t* test. **B** Immunoblotting of SLC26A3 in DLD1 and HCT116 cells after treatment with lactic acid. **C** Immunofluorescence of SLC26A3 in HCT116 cells under lactate or non-lactate treatment. Scale bars: 50 μm. **D** Co-localization of SLC26A3 with membrane markers (EPCAM) and cytosolic markers (Pan-Keratin) in HCT116 cells. Scale bars: 20 μm. **E** The lactated modification of SLC26A3 is detected by co-IP assay in lactate or non-lactate treated groups. **F** Predict the lactylation sites of SLC26A3 using the MOE software and visualize the results on the PDB website (www.rcsb.org). **G** Mass spectrometry revealed that SLC26A3 is lactylated at K536. **H** Mutation of the K536 site attenuates SLC26A3 lactylation. **I** Immunoblotting to detect SLC26A3 expression after lactate or non-lactate treatment, and A485 or non-A485 treatment. Quantified immunoblotting results using ImageJ. Two-way anova test. **J** A ubiquitinated lysine residue (K449) in SLC26A3 predicted by UniProt online database (www.uniprot.org). **K** Lactate treatment increases the ubiquitination level of SLC26A3. *P < 0.05, **P < 0.01, and ***P < 0.001, Data are representative of three independent experiments.

As mentioned earlier, acidic environment affects the expression level of SLC26A3. Given that lactate is a major component contributing to extracellular acidity, we sought to further investigate whether lactate itself influences SLC26A3 expression. The metabolic pattern of tumor tissue significantly differs from that of normal tissue. Due to the Warburg Effect, tumor tissues exhibit higher levels of lactate. Lactate dehydrogenase A (LDHA) is a key factor in the lactate metabolism process. Immunohistochemical results showed that the expression level of LDHA was higher in tumor tissues (Fig. 3A), indicating lactate accumulation in the tumor tissue. To verify whether high lactate levels affect the expression of SLC26A3, we added lactic acid to the CRC cell culture medium, and SLC26A3 expression was found to be lower than that in the control group (Fig. 3B). Cell immunofluorescence experiments also confirmed that lactic acid treatment reduced SLC26A3 levels (Fig. 3C). Moreover, we also examined how lactate influences the subcellular localization of SLC26A3 (Fig. 3D and Supplementary Fig. S2B), as well as the baseline subcellular distribution of SLC26A3 in normal intestinal epithelial cells (Supplementary Fig. S2C). The results still demonstrate that SLC26A3 is primarily localized to the plasma membrane, and lactate reduces its membrane expression level. Although lactate has been shown to regulate target protein functions via lactylation, the precise mechanism by which it modulates the expression or stability of SLC26A3 remains unclear. After treatment of the cells with lactic acid, we detected the lactylation levels of SLC26A3 via a pan-lactylation antibody and found that lactylation of SLC26A3 was promoted (Fig. 3E). Using Molecular Operating Environment (MOE) software, specific sites of SLC26A3 lactylation were predicted, revealing that the B464 and B521 lysine residues can undergo lactylation (Fig. 3F). This suggests that certain lysine residues of SLC26A3 are potentially subject to lactylation. We further validated by mass spectrometry that SLC26A3 undergoes lactylation at K536 (Fig. 3G). Then we transfected cells with SLC26A3 K536R mutant plasmids to verify the mass-spectrometry results. After lactate treatment, the WT still showed SLC26A3 lactylation, whereas lactylation was abolished in the K536R mutant, demonstrating that K536 is the lactylation site on SLC26A3 (Fig. 3H). To determine whether the reduced expression of SLC26A3 is linked to its lactylation, we examined the relationship between its stability and lactate. The stability of the SLC26A3 protein was assessed via cycloheximide (CHX) chase assays, which revealed that SLC26A3 protein stability decreased after acidic culture or lactic acid treatment. The acetyltransferase p300 inhibitor A485, which is known to reduce lactylation levels [19], was added along with lactate or treatment under pH7.4/6.8 and partially restored SLC26A3 protein stability (Fig. 3I and Supplementary Fig. S2A). The results indicate that SLC26A3 undergoes lactylation in an acidic environment, reducing its stability and thus decreasing it expression. We used the UniProt online database to identify a ubiquitinated lysine residue (K449) in SLC26A3 that lies adjacent to our previously predicted lactylation site (K536), suggesting potential crosstalk between lactylation and ubiquitination (Fig. 3J). We then established a lactate-treatment group and a control group to examine whether lactate affects SLC26A3 ubiquitination. Cells were pre-treated with 10 µM MG-132 (26S proteasome inhibitor) for 2 h before lactate exposure. Compared with the control, lactate markedly increased the ubiquitin signal on SLC26A3 (Fig. 3K), indicating that lactylation may act as a “degron” that recruits the relevant E3 ligase to ubiquitinate SLC26A3, thereby enhancing its degradation via the ubiquitin-proteasome pathway.

### Low SLC26A3 levels drive stemness of tumor cells

**Fig. 4:**
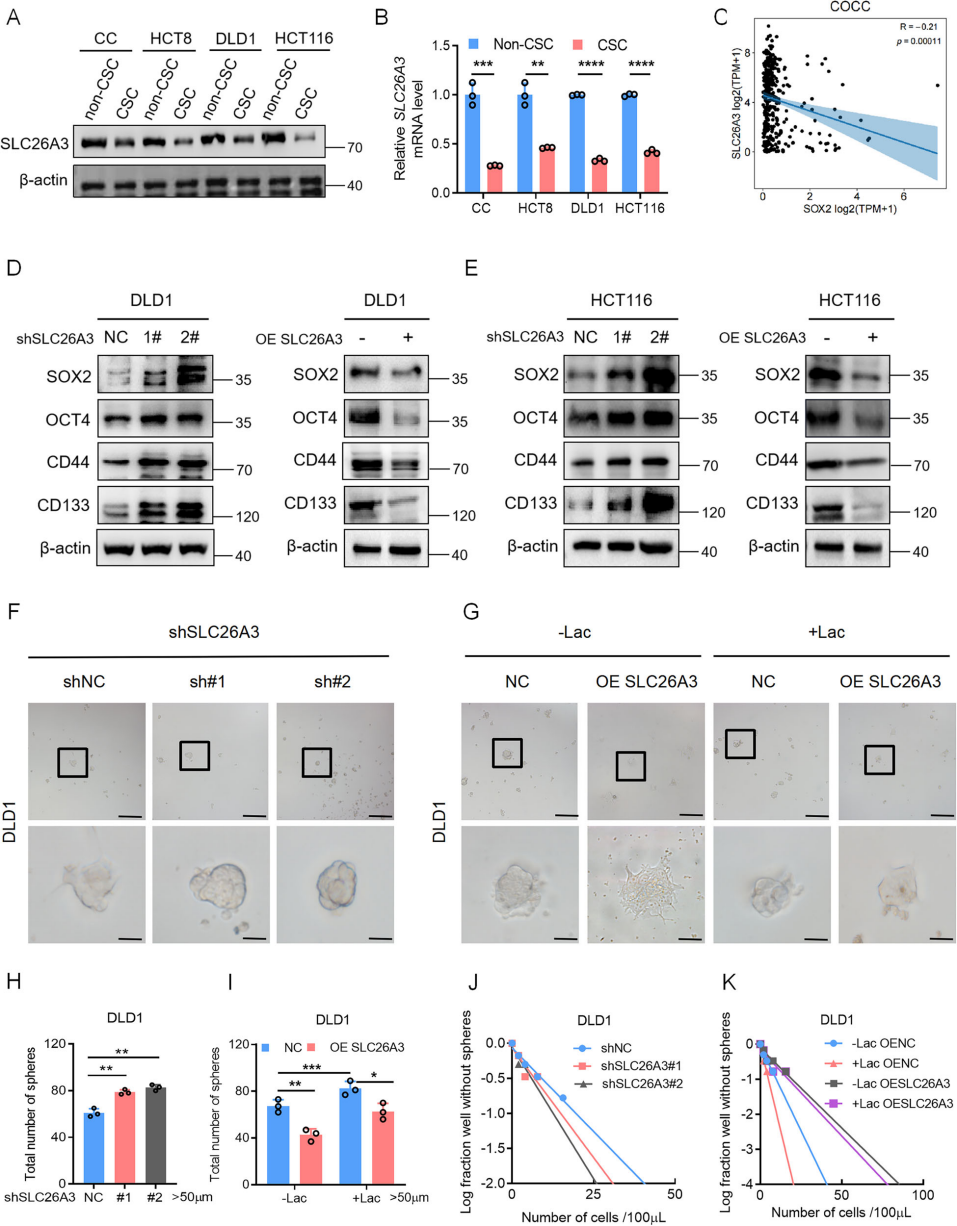
Low SLC26A3 levels exacerbate stemness and malignant phenotype of tumor cells. **A**, **B** Immunoblotting and qPCR of SLC26A3 in CSC and non-CSC from different CRC cell lines. Student’s *t* test. **C** SLC26A3 is negatively correlated with the expression of stemness marker SOX2 in COCC database. Pearson correlation analysis. **D**, **E** Immunoblotting of stemness markers in DLD1 and HCT116 cells that overexpress SLC26A3 or treated with SLC26A3-targeting shRNA. Representative images (**F**) and quantified data (**H**) for tumor spheres (with diameters larger than 50 µm) formed by control and SLC26A3-targeting shRNA treated DLD1 cells. Scale bars: upper, 200 μm; lower, 50 μm. Student’s *t* test. Representative images (**G**) and quantified data (**I**) for tumor spheres (with diameters larger than 50 µm) formed by control and SLC26A3-overexpress DLD1 cells. Scale bars: upper, 200μm; lower, 50μm. Student’s *t* test. Limiting dilution assay of DLD1 cells treated with SLC26A3-targeting shRNA (**J**) or SLC26A3-overexpress (**K**). *P < 0.05, **P < 0.01, and ***P < 0.001, Data are representative of three independent experiments.

Bioinformatic analyses have indicated that SLC26A3 expression is associated with stemness. Therefore, we next sought to validate in vitro how SLC26A3 influences the stemness of tumor cells. We found that SLC26A3 expression was lower in CRC stem cells than in non-stem cells (Fig. 4A, B). To investigate the effect of SLC26A3 on the stemness phenotype of CRC cells, we analyzed the COCC and TCGA databases, which revealed that SLC26A3 expression was negatively correlated with the expression of several stemness markers (Fig. 4C and Supplementary Fig. S3A, B). SLC26A3 knockdown resulted in increased expression of stemness markers, whereas overexpression of SLC26A3 led to decreased expression (Fig. 4D, E and Supplementary Fig. S3C, D). Tumor sphere formation assays indicated that SLC26A3 knockdown promoted the sphere formation of tumor cells (Fig. 4F, H and Supplementary Fig. S3E, G); conversely, SLC26A3 overexpression suppressed sphere formation. However, when SLC26A3-overexpressing cells were cultured with lactate, their sphere-forming ability partially recovered (Fig. 4G, I and Supplementary Fig. S3F, H). Additionally, SLC26A3 knockdown increased the self-renewal capacity of tumor cells (Fig. 4J and Supplementary Fig. S3I); overexpression of SLC26A3 reduced the self-renewal capacity, but this capacity was partially restored under lactate conditions (Fig. 4K and Supplementary Fig. S3J). Simulating the acidic tumor microenvironment instead of lactate treatment yielded similar results after repeated experiments (Supplementary Fig. S4A–F).These results indicate that low SLC26A3 expression promotes CRC stemness.

### Low SLC26A3 expression leads to chemotherapy resistance and enhances invasion and migration of CRC cells

**Fig. 5:**
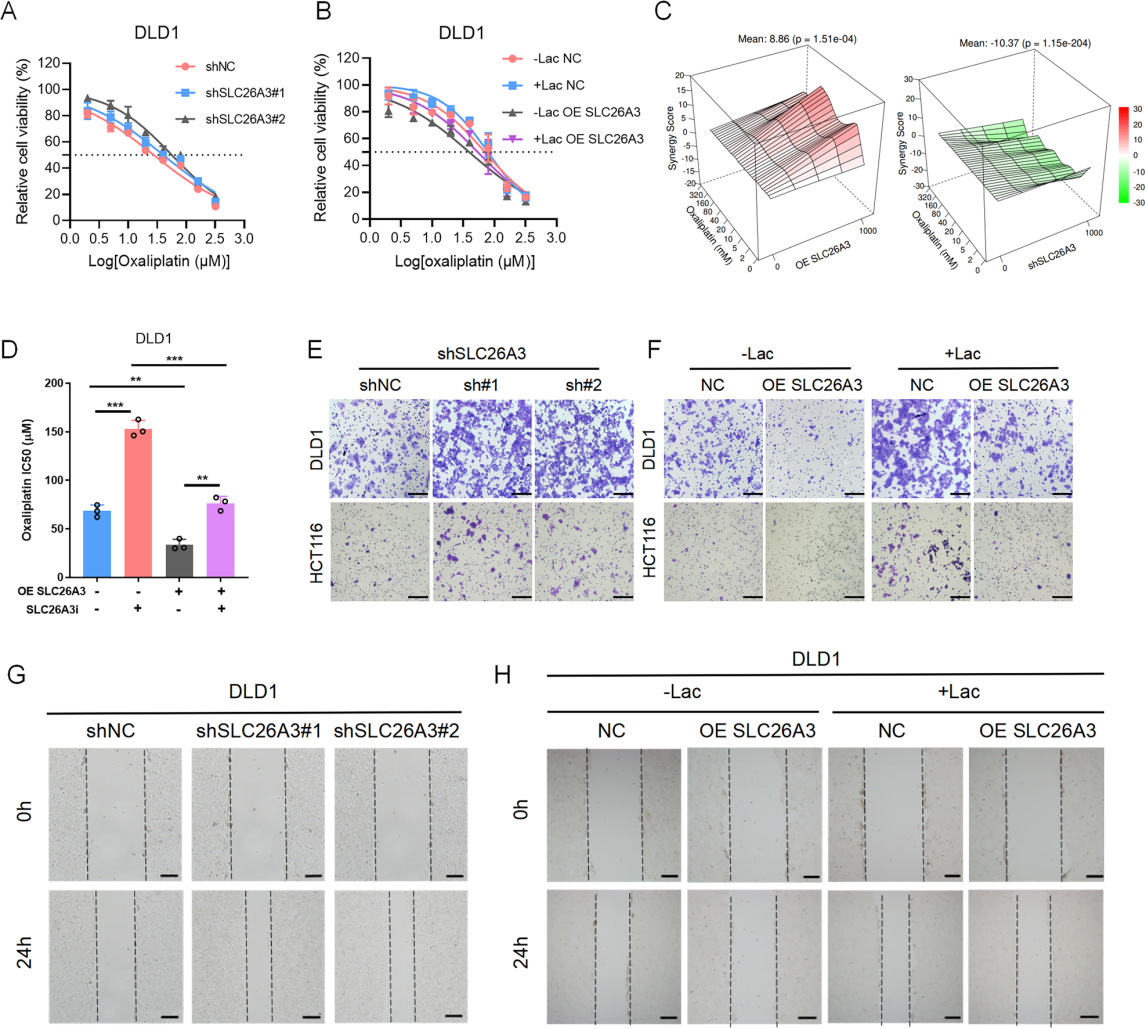
Low SLC26A3 expression leads to chemotherapy resistance, invasion and migration of CRC Cells. **A** Cell viability of control and SLC26A3-knockdown DLD1 cells with OXA treatment. The IC50 is shown as a dotted line. **B** Cell viability of control and SLC26A3-overexpress DLD1 cells with OXA and lactate treatment. The IC50 is shown as a dotted line. **C** Bliss synergistic analysis (www.synergyfinder.org) shows that overexpression of SLC26A3 has synergistic effect with OXA, and knockdown SLC26A3 has antagonistic effect with OXA in DLD1 cells. **D** Cell viability of control and SLC26A3-overexpress DLD1 cells with SLC26A3 inhibitor. Student’s *t* test. **E** Representative images of the transwell assay. Scale bars: 100 μm. **F** Representative images of the transwell assay with lactate treatment. Scale bars: 100 μm. **G** Representative images of the wound-healing assay. Scale bars: 50 μm. **H** Representative images of the wound-healing assay with lactate treatment. Scale bars: 50 μm. *P < 0.05, **P < 0.01, and ***P < 0.001, Data are representative of three independent experiments.

Cancer stem cells often enhance their drug resistance through mechanisms including enhanced drug efflux and DNA repair, metabolic reprogramming, or epigenetic regulation. We analyzed whether SLC26A3 expression influenced the resistance of HCT116 and DLD1 CRC cells to oxaliplatin (OXA). The expression of SLC26A3 was decreased in OXA-resistant HCT116 and DLD1 cell (Supplementary Fig. S5A). SLC26A3 knockdown increased the half maximal inhibitory concentration (IC50) of OXA while overexpression of SLC26A3 decreased that. But when SLC26A3-overexpressing cells were cultured with lactate, the IC50 was partially recovered (Fig. 5A, B and Supplementary Fig. S5D–G). BLISS synergy analysis indicated that SLC26A3 overexpression synergized with OXA treatment, while SLC26A3 knockdown antagonized the effect of OXA (Fig. 5C and Supplementary Fig. S5C). Treatment of CRC cells with SLC26A3 inhibitor increased the IC50 of OXA (Fig. 5D and Supplementary Fig. S5B). Similar results were obtained in resistance assays using 5-FU (Supplementary Fig. S5H–N).

Similarly, we analyzed the impact of SLC26A3 on invasion and migration abilities of CRC cells. The results revealed that low SLC26A3 expression promoted tumor cell invasion and migration, whereas SLC26A3 overexpression had the opposite effect. Treatment of tumor cells with lactate promoted invasion (Fig. 5E, F and Supplementary Fig. S6A, B) and migration (Fig. 5G, H and Supplementary Fig. S6C–H), and similar results were obtained under acidic conditions (Supplementary Fig. S7A–F). In summary, these findings suggest that SLC26A3 acts as a tumor suppressor in CRC, inhibiting the malignant biological behavior of CRC cells.

### SLC26A3 interacts with HuR/CUGBP1 and regulates expression of genes related to malignant phenotype of tumor cells

**Fig. 6:**
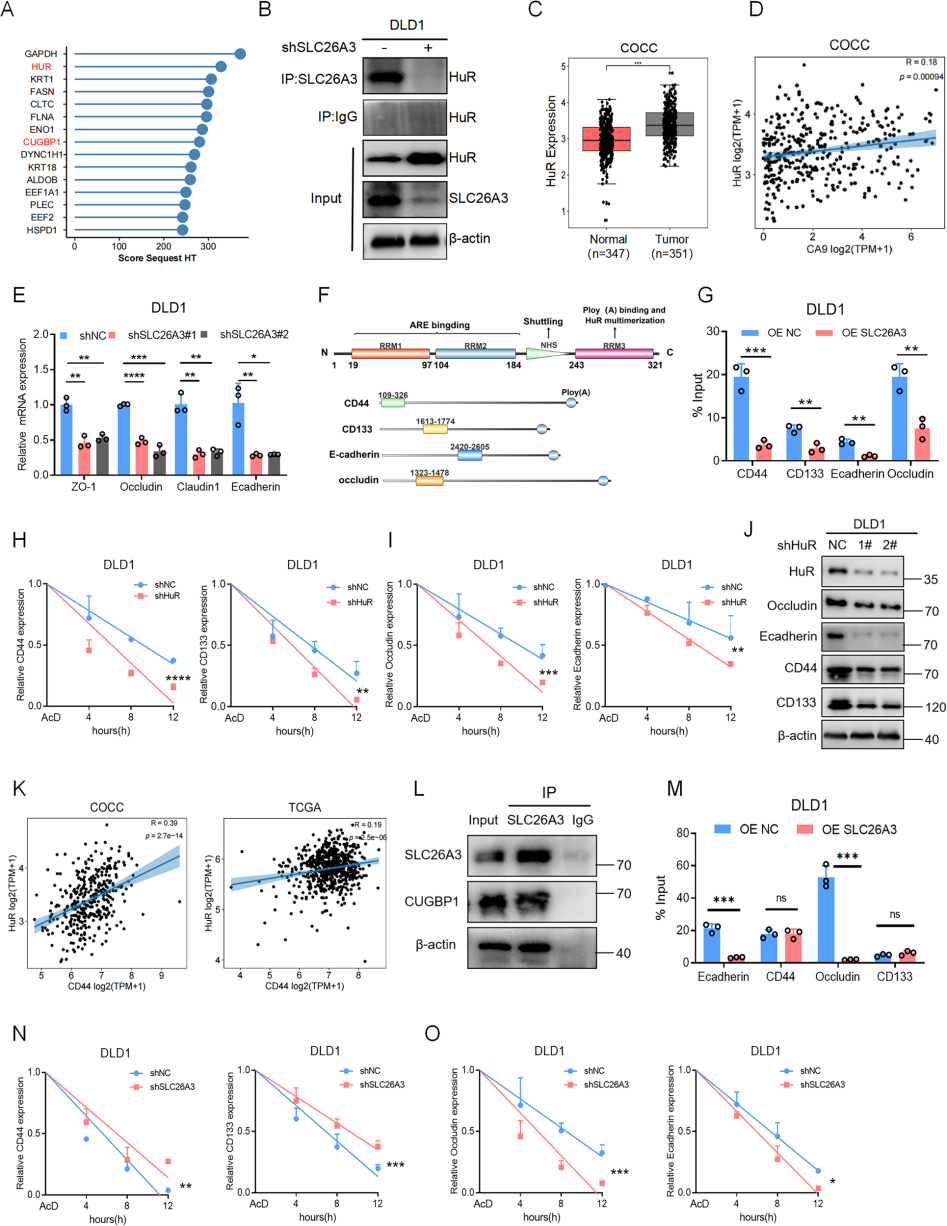
SLC26A3 interacts with HuR/CUGBP1 and regulates the expression of genes related to malignant phenotype of tumor cells. **A** IP-MS results revealed key proteins interacting with SLC26A3. **B** co-IP assay shows that there is protein interaction between SLC26A3 and HuR. **C** Analysis of the COCC database indicates that HuR is highly expressed in CRC. Wilcox rank sum test. **D** Analysis of the COCC database indicates that HuR expression is positively correlated with CA9 expression in CRC. Pearson correlation analysis. **E** qPCR of some epithelial phenotypic after SLC26A3 knockdown in DLD1 cells. Student’s *t* test. **F** The binding degree between HuR and related mRNA in control and overexpressing group in DLD1 cells is detected by RIP assay, and the immunoprecipitated RNA is quantified by qPCR. Student’s *t* test. **G** Schematic diagram of HuR functional region structure, and relative mRNA fragments binded with it. **H**, **I** qPCR of related mRNA after ActD treatment in control and HuR knock-down group in DLD1 cells. Two-way anova test. **J** Immunoblotting of related marker after knockdown of HuR in DLD1 cells. **K** Analysis of the COCC and TCGA database indicates that HuR expression is positively correlated with expression of stemness mark CD44 in CRC. Pearson correlation analysis. **L** co-IP assay shows that there is protein interaction between SLC26A3 and CUGBP1. **M** The binding degree between HuR and related mRNA in control and overexpressing group in DLD1 cells is detected by RIP assay, and the immunoprecipitated RNA is quantified by qPCR. Student’s *t* test. **N**, **O** qPCR of related mRNA after ActD treatment in control and SLC26A3 knock-down group in DLD1 cells. Two-way anova test. *P < 0.05, **P < 0.01, and ***P < 0.001, Data are representative of three independent experiments.

To uncover the molecular mechanism by which SLC26A3 restrains oncogenic programs, we interrogated its protein-protein interaction network. Immunoprecipitation followed by mass spectrometry (IP-MS) analysis was subsequently conducted to identify proteins that interact with SLC26A3 (Fig. 6A and Supplementary Fig. S8A, B). Among the proteins primarily bound by SLC26A3, only HuR and CUGBP1 were RNA-binding proteins. HuR is one of the most studied RNA post-transcriptional regulators [20, 21]. It is overexpressed in various human cancers, and this phenotype indicates poor clinical outcomes; HuR promotes tumorigenesis by interacting with a subset of oncogenic mRNAs involved in different cancer characteristics and treatment resistance [22, 23]. CUGBP1 can regulate the expression of oncogenes or tumor suppressor genes through its interactions with mRNAs to affect their stability and translation, which in turn influences cell proliferation and survival [24, 25]. Immunoblotting confirmed that SLC26A3 interacts with HuR, with enhanced binding upon SLC26A3 overexpression and reduced binding upon knockdown (Fig. 6B and Supplementary Fig. S8C). Immunofluorescence also revealed their co-localization (Supplementary Fig. S8D). HuR is a key factor in promoting cancer progression and is highly expressed in CRC (Fig. 6C and Supplementary Fig. S8E). Moreover, its expression is positively correlated with the expression of the acidic marker CA9 (Fig. 6D and Supplementary Fig. S8F). Pathway-based GSEA (Gene Set Enrichment Analysis) revealed that SLC26A3 is related to the expression of epithelial junction proteins and EMT (Supplementary Fig. S8H). We then found that SLC26A3 knockdown reduced the expression of the junction proteins ZO-1, Occludin, Claudin1, and E-cadherin, which are associated with cell migration and EMT (Fig. 6E). As mentioned earlier, SLC26A3 knockdown increased the expression of the stemness markers OCT4, CD44, SOX2, and CD133 (Fig. 4D). Given that SLC26A3 interacts with the RNA-binding protein HuR, we hypothesized that changes in SLC26A3 expression levels affect the mRNA levels of oncogenes through HuR, thereby affecting their protein expression.

HuR contains three RNA recognition motifs (RRMs) [23], and we predicted the HuR-binding regions in the above eight mRNAs via the online tool JASPAR and designed corresponding primers. RIP experiments revealed that HuR can bind to the mRNAs of CD44, CD133, Occludin, and E-cadherin, and this binding was weaker when SLC26A3 was overexpressed (Fig. 6F, G). Next, we explored how HuR affects mRNA levels. CRC cells with HuR knockdown were treated with actinomycin D (ActD), and we found that the mRNA stability of CD44, CD133, Occludin and E-cadherin decreased (Fig. 6H, I). Western blotting revealed that HuR knockdown reduced their protein expression (Fig. 6J). The analyses of the COCC and TCGA databases also revealed that HuR expression is positively correlated with the expression of stemness markers (Fig. 6K and Supplementary Fig. S8G). The above results indicated that HuR stabilized these mRNAs.

Studies have shown that CUGBP1 competes with HuR for binding to mRNAs [26]. Immunoprecipitation followed by mass spectrometry and immunoblotting analysis revealed that SLC26A3 also interactd with CUGBP1 (Fig. 6L and Supplementary Fig. S8I). Previously, we demonstrated that HuR bound to the mRNAs of CD44, CD133, Occludin, and E-cadherin. We then detected that CUGBP1 could only bind to Occludin and E-cadherin and reduced their stability (Fig. 6M and Supplementary Fig. S8J–L). We assessed the mRNA stability in CRC cells with SLC26A3 knockdown, the results showed that the stability of CD44 and CD133 increased (Fig. 6N) but Occludin and E-cadherin decreased (Fig. 6O). The reason for this phenomenon is CUGBP1 has a stronger ability to bind to Occludin and E-cadherin than HuR, ultimately leading to a decrease in their stability. In summary, SLC26A3 interacts with HuR/CUGBP1. When SLC26A3 expression is downregulated, its binding to HuR/CUGBP1 weakens, leading to enhanced binding of HuR/CUGBP1 to downstream oncogenic mRNA subsets, which affects their stability and promotes tumor progression.

### Normalization of tumor acidic microenvironment and induction of SLC26A3 expression inhibit CRC development

**Fig. 7:**
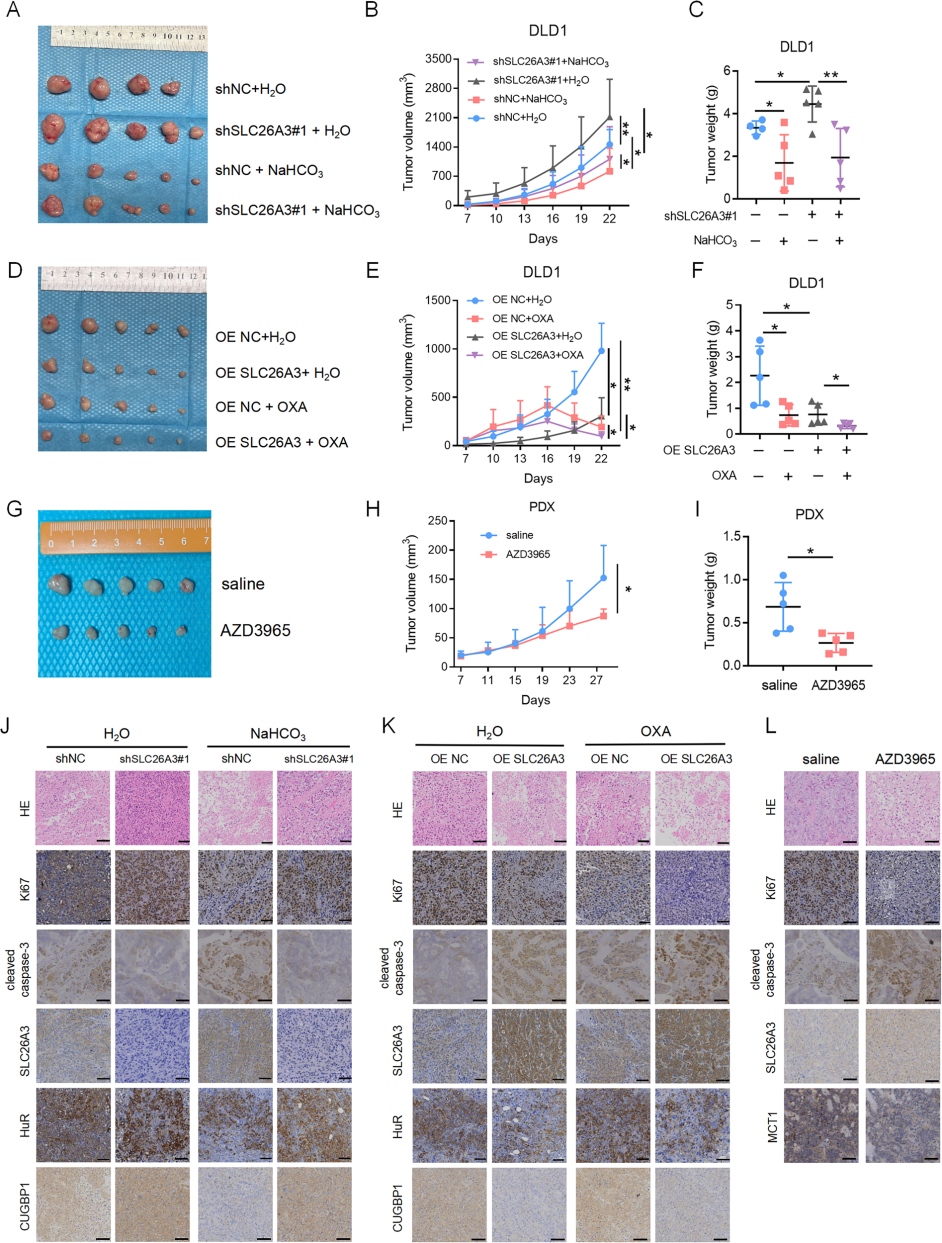
Normalization of tumor acidic microenvironment and induction of SLC26A3 expression inhibit CRC development. Tumor images (**A**), tumor volumes (**B**) and tumor weights (**C**) of mice (*n* = 5) subcutaneously injected with control and SLC26A3-knockdown DLD1 cells and subsequently treated with water or 200 mM sodium bicarbonate (NaHCO_3_). Two-way anova test. Tumor images (**D**), tumor volumes (**E**) and tumor weights (**F**) of mice (*n* = 5) subcutaneously injected with control and SLC26A3 overexpressing DLD1 cells and subsequently treated with water or 10 mg/kg OXA. Two-way anova test. Tumor images (**G**), tumor volumes (**H**) and tumor weights (**I**) of humanized tumor xenograft mice (*n* = 5) treated with water or 50 mg/kg AZD3965. Two-way anova test. **J** Immunohistochemical staining of tumors from NaHCO_3_/water treated mice. Scale bars: 50 μm. **K** Immunohistochemical staining of tumors from OXA/water treated mice. Scale bars: 50 μm. **L** Immunohistochemical staining of tumors from saline/AZD3965-treated mice. Scale bars: 50 μm. *P < 0.05, **P < 0.01, and ***P < 0.001, Data are representative of three independent experiments.

**Fig. 8:**
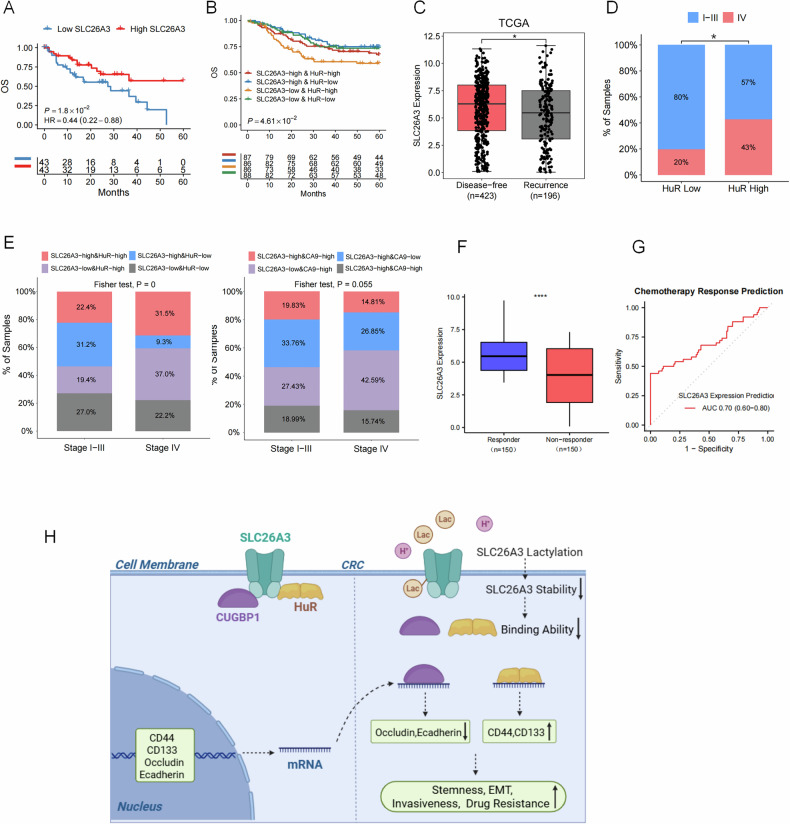
SLC26A3 and HuR expression associated with prognosis of CRC patients. **A** Overall survival of IV stage CRC patients in SLC26A3 low/high expression group in COCC database. Log-rank test. **B** Overall survival of CRC patients in SLC26A3 low/high expression group and HuR low/high expression group in COCC database. Kaplan–Meier method and Log-rank test. **C** SLC26A3 expression in disease-free and recurrence CRC patients in TCGA database. Wilcox rank sum test. **D** Percentage of stage I-III and stage IV CRC patients in HuR low/high expression group in COCC database. Chi-Squared test. **E** Percentage of SLC26A3 low/high expression and HuR or CA9 low/high expression patients in stage I-III group and IV group in COCC database. Fisher’s exact test. **F** Patients’ responsiveness to chemotherapy is positively correlated with SLC26A3 expression levels in COCC database. **G** Evaluation of the chemotherapy sensitivity prediction model based on SLC26A3 expression in COCC database. **H** In the acidic tumor microenvironment, lactylation of SLC26A3 precipitates its destabilization, impairing binding to HuR/CUGBP1 and subsequently diminishing their regulation of downstream mRNA stability. This molecular cascade modulates tumor cell stemness, EMT, invasiveness, and drug resistance phenotypes. *P < 0.05, **P < 0.01, and ***P < 0.001.

SLC26A3 expression and tumor acidic microenvironment are associated with tumor progression. To determine whether altering the acidic tumor microenvironment and SLC26A3 expression can suppress CRC growth, CRC cells were injected subcutaneously into nude mice, and water or sodium bicarbonate (NaHCO_3_) solution was injected into the tumor [27, 28]. We found that SLC26A3 knockdown promoted tumor progression and that NaHCO_3_ treatment effectively mitigated the tumor progression induced by SLC26A3 downregulation (Fig. 7A–C). The overexpression of SLC26A3 inhibited tumor growth, and simultaneous OXA injection and SLC26A3 overexpression further suppressed tumor progression (Fig. 7D–F). Treatment of mice with the selective MCT1 inhibitor AZD3965 inhibited lactate accumulation within the tumor, and the results showed that tumors in AZD3965-treated mice were significantly reduced (Fig. 7G–I). Immunohistochemical staining of mouse tumor tissue sections revealed that expression of SLC26A3 was downregulated during tumor progression, whereas expression of HuR and CUGBP1 was upregulated (Fig. 7J, K). AZD3965 treatment suppressed tumor progression while upregulating SLC26A3 expression (Fig. 7L). Additionally, we found that patients with relatively high SLC26A3 expression in stage IV CRC had longer overall survival (Fig. 8A). Patients with low SLC26A3 expression and high HuR expression had the worst prognosis (Fig. 8B). CRC patients with relatively low SLC26A3 expression were more prone to recurrence (Fig. 8C). Patients with high HuR and CA9 expression and low SLC26A3 expression were more likely to develop distant metastasis (Fig. 8D, E). In the COCC cohort, patients’ responsiveness to chemotherapy was positively correlated with SLC26A3 expression levels (Fig. 8F). Therefore, SLC26A3 may serve as a potential biomarker for chemotherapy sensitivity (Fig. 8G). These findings suggest that reducing tumor microenvironment acidity and inducing SLC26A3 expression can inhibit CRC development and provide new theoretical support for the clinical treatment of CRC.

## Discussion

Acidity is a key physicochemical characteristic of the tumor microenvironment. Due to the high metabolic activity of tumor tissues, relative insufficiency of tissue perfusion, and accumulation of metabolic byproducts, the tumor microenvironment often becomes acidic and hypoxic. The acidity of the tumor microenvironment is primarily formed by the accumulation of lactate from metabolic processes, enhanced aerobic glycolysis due to hypoxia, and the secretion of hydrogen ions by tumor cells [29]. The acidic tumor microenvironment drives tumorigenesis by promoting cellular adaptations that enhance tumor cell survival and aggressiveness [30]. The acidic tumor microenvironment influences tumor stem cell phenotype, tumor cell invasion, migration, and every stage of cancer development by participating in a complex network of interactions, including epigenetics, cellular metabolism, cell proliferation, and tumor immune evasion [31–33]. Transcriptomic studies have shown that the acidic microenvironment can regulate gene expression at both transcriptional and post-transcriptional levels in vitro. For instance, highly acidic phosphates can lead to double-stranded DNA breaks and inhibit the repair of damaged DNA, resulting in the accumulation of abnormal chromosomes [34, 35]. Notably, the acidic tumor microenvironment can also affect tumor progression by influencing membrane ion channel proteins.Research has shown that an extracellular acidic environment can stimulate EMT because acid-induced activation of acid-sensing ion channels increases intracellular Ca^2+^ concentration, leading to the loss of polarity and intercellular adhesion in epithelial cells, thus promoting EMT [36]. The acidic microenvironment promotes tumor progression by activating acid-sensing ion channel 1, inducing IL-8 expression, and activating matrix metalloproteinases-2/-9 [37]. Lactylation was first reported in 2019 by Zhang et al. [14]. They identified lactate as a novel post-translational modification molecule through mass spectrometry, binding to specific amino acid residues on proteins via ester or covalent bonds. This study revealed that lactate is not only a metabolic product but also regulates protein structure and function, thereby affecting biological processes in cells. Collectively, these studies demonstrate that the acidic microenvironment induces proteomic alterations, which in turn contribute to tumor initiation and progression. As explored in this paper, acidic conditions or the addition of lactate lead to the lactylation modification of SLC26A3, resulting in decreased stability. The underlying mechanism is as follows: SLC26A3 contains 10-14 transmembrane α-helices, with both the N- and C-termini facing the cytoplasmic side [38]. K536 is positioned in the intracellular loop between transmembrane helices 11 and 12, and its side chain is fully exposed to the cytosolic side. Lactylation of SLC26A3 occurs on the cytosolic side, catalyzed by p300/CBP using lactyl-CoA as the donor, with K536 being the primary site. This modification introduces a negative charge that loosens the protein conformation, enabling recognition by the E3 ligase TRIM21 and subsequent polyubiquitination and proteasomal degradation. Consequently, membrane localization of SLC26A3 is reduced, extracellular acidification is intensified, and a positive feedback loop of “lactylation–degradation–increased acidity” is established. The downregulation of SLC26A3 expression leads to increased expression of stemness phenotype proteins and EMT-related proteins in tumor cells, thereby promoting tumor progression.

The solute carrier gene (SLC) super-family encodes membrane-bound transport proteins, consisting of 55 gene families that encode 456 membrane proteins, of which at least 362 are predicted to have functional roles. The gene products include passive transporters, symporters, and antiporters. They transport a wide variety of substrates, including inorganic ions, amino acids or oligopeptides, sugars, metal cations, bile salts, carboxylates, and other organic anions [39]. SLC transport proteins are widely distributed and abundant in the body, and beyond their basic transport functions, they play other important roles. Some members also exhibit tumor suppressor activity: four transporters, SLC5A8, SLC26A3, SLC39A1, and SLC22A18, have been identified as tumor suppressors, with SLC26A3’s tumor-suppressive function potentially related to changes in intracellular and extracellular pH [17]. SLC26A3 expression is significantly reduced in CRC cell lines, and its downregulation is associated with CRC progression. However, the reasons for SLC26A3 downregulation in CRC and its relationship with CRC progression have not been thoroughly studied. Previous studies have shown that SLC26A3 is mainly expressed on the luminal side of colon epithelial cells and functions primarily as a Cl^-^/HCO_3_^-^ exchanger [40, 41]. It has been suggested that when the concentrations of H^+^ and HCO_3_^-^ in the tumor microenvironment are significantly altered, tumor cells adjust the activity and expression levels of ion channels and transporters to better maintain intracellular pH and HCO_3_^-^ concentration [42]. Our experiments also support this, as CRC cells cultured in acidic medium expressed lower levels of SLC26A3 compared with those cultured in alkaline conditions. Knockout of SLC26A3 lowers extracellular HCO_3_^-^ levels and aggravates acidification. The acidic tumor microenvironment facilitates immune evasion, enhances invasion and metastasis, induces therapeutic resistance, and reshapes metabolic ecology, while interacting with the stroma and vasculature, thereby driving the tumor toward a more malignant phenotype. That fully consistent with our in vivo and in vitro findings. Our subsequent research demonstrated that SLC26A3 acts as a tumor suppressor; its low expression promotes stemness, invasion, and migration abilities in CRC cells, while increasing sensitivity to OXA. Enhanced cancer-cell stemness promotes chemoresistance by jointly upregulating ABC transporters (ABCB1, ABCG2, ABCC1) to efflux drugs, activating DNA-repair pathways through stemness-maintenance factors (SOX2, OCT4, NANOG), and increasing anti-apoptotic proteins via stemness-associated signaling [43, 44].

Research has shown that the ability of SLC26A3 to inhibit cancer cell proliferation is unrelated to its transport function; even SLC26A3 without transport activity can suppress cancer cell growth [45], indicating that SLC26A3 does not act alone in exerting its tumor-suppressive effects. Recent studies reported that HuR/CUGBP1 play important roles in maintaining intestinal epithelial integrity [16]. HuR and CUGBP1 are widely expressed proteins that regulate various cell functions, including tumor progression, proliferation, and apoptosis, by acting on the non-coding regions of target mRNAs [24, 25]. We demonstrated the interaction between SLC26A3 and HuR/CUGBP1. HuR can bind to the mRNAs of stemness genes CD44, CD133, tight junction protein Occludin, and EMT-related gene E-cadherin. CUGBP1 competitively binds to the mRNAs of Occludin and E-cadherin. After knocking down SLC26A3, the mRNA stability of CD44 and CD133 increases, while the mRNA stability of Occludin and E-cadherin decreases. This is due to the reduced expression of SLC26A3 leading to decreased binding with HuR/CUGBP1, which in turn results in increased binding of HuR/CUGBP1 to the aforementioned mRNAs, thereby regulating their stability. HuR enhances the mRNA stability of CD44, CD133, Occludin, and E-cadherin, but competitive binding by CUGBP1 ultimately reduces their stability. We speculate that CUGBP1 has an opposing effect to HuR, weakening mRNA stability, and has a stronger competitive binding capacity when both are present.

Beyond tumor cell-intrinsic alterations, the gut microbiota is increasingly recognized as a key modulator of CRC progression through metabolites that reshape the tumor microenvironment. Microbiota-derived lactate and short-chain fatty acids can directly alter extracellular pH and lactate levels, thereby potentially influencing the lactylation landscape of host proteins. Although our study focused on the intrinsic regulation of SLC26A3 lactylation, we speculate that specific microbial communities or their metabolites may exacerbate or attenuate this modification. Future studies integrating multi-omics profiling of the microbiome with quantitative lactyl-proteomics will clarify whether microbiota-derived signals cooperate with acidic stress to fine-tune SLC26A3 stability and its downstream RNA-binding protein network, thereby providing a more holistic view of CRC pathogenesis [46].

In summary, the acidic microenvironment-SLC26A3-HuR/CUGBP1 axis regulates the stability of oncogene mRNAs, modulating their expression levels and ultimately promoting stemness, invasion, migration, EMT, and drug resistance in tumor cells. This study proposes two translational strategies-neutralizing the intratumoral microenvironment or genetically restoring SLC26A3 expression, and using MCT1 inhibitors to block metabolism. Pre-clinical models confirm that both approaches synergistically enhance oxaliplatin efficacy and suppress metastasis, offering immediately testable combination regimens and predictive biomarkers for clinical application.

## Materials and methods

### Cells and specimens

Human immortalized colon epithelial cells and CRC cells lines were obtained at Professor Lan Ping’s Research Group, The Sixth Affiliated Hospital of Sun Yat-Sen University. Human CRC stem cells (HCT116, DLD1, HCT8, and CC) were obtained from previous studies [11]. The CRC specimens used in the experiments were obtained from The Sixth Affiliated Hospital of Sun Yat-Sen University and obtained after patients provided informed consent.

### Single-cell analysis

The single-cell transcriptome data (GSE178341) used in this study was sourced from the GEO database (https://www.ncbi.nlm.nih.gov/geo/). Original authors had performed initial quality control on this dataset. Gene expression data from all cells underwent log-normalization using the “LogNormalize” function (scale.factor = 10,000). The top 2000 highly variable genes were identified for downstream dimensionality reduction. During scaling, regression correction was applied via the “var.to.regress” parameter to account for mitochondrial gene content percentage. Principal component analysis (PCA) was employed for linear dimensionality reduction, followed by preliminary cell clustering and annotation using the “FindClusters” function with 50 principal components (resolution = 0.8). Nonlinear dimensionality reduction and visualization of cell clusters were achieved through UMAP. Secondary clustering combined with copy number variation analysis (inferCNV) was implemented to identify malignant cells within epithelial populations. Phenotypic scores were calculated using the “singscore” function from the “irGSEA” package.

### Acidic culture conditions

HEPES (25 mM) and PIPES (25 mM) (Sigma-Aldrich, St. Louis, MO,USA) were added to RPMI1640 or DMEM/F12 (Thermo, Waltham, MA, USA) serum-free medium containing 10% FBS (Thermo) and 1% penicillin-streptomycin (Thermo), and the pH was titrated to 7.4/6.8 using 1 M NaOH or 1 M HCl (GHTECH, Dongguan, China). After incubating the medium for 24 h, the pH was re-titrated [11].

### Immunofluorescence

The culture medium was washed with 1× PBS (Servicebio, Wuhan, China), then fixed in pre-cooled 4% PFA (Biosharp, Hefei, China) at 4 °C for 20 min. The samples were blocked at room temperature for 1 h in PBS containing 0.5% NGS (Thermo) and 0.2% Triton-X100 (Sigma). The primary antibody was incubated at room temperature for 1.5–2 h or overnight at 4 °C. The samples were washed with PBS, then incubated with the secondary antibody at room temperature for 1.5–2 h. After washing with PBS, the samples were stained with DAPI (Servicebio) at room temperature for 3–5 min, followed by a PBS wash. The slides were mounted with anti-fade mounting medium and air-dried [47]. Images were captured using ZEISS confocal microscope and processed using ZEISS 3.8 software. The antibodies used included anti-SLC26A3 (Abcam, Waltham, MA, USA), anti-CA9 (Abcam), fluorescence-conjugated secondary antibodies (Life Technologies, Carlsbad, CA, USA). Confocal fluorescence microscopy was performed on a Zeiss LSM 880 Airyscan microscope equipped with 405, 488, 561 and 633 nm laser lines. Images were acquired with a Plan-Apochromat 63×/1.40-NA oil-immersion objective at 1024 × 1024 pixel resolution. Z-stacks (0.13 µm optical sections, 1× digital zoom) were recorded with the Airyscan detector in super-resolution mode. Laser power, gain and offset were kept constant for all samples within one experiment (typically 2% 488 nm, 1.5% 561 nm, gain700–800). Raw Airyscan images were processed automatically in ZEN 3.4 (Airyscan processing strength = 6). Maximum-intensity projections and colocalisation analysis (Pearson coefficient, Manders overlap) were performed in ImageJ (Fiji) with the JACoP plugin. All representative images shown are single optical sections unless stated otherwise.

### Immunoblotting

Cells and tissues were lysed in Western and IP lysis (Beyotime, Shanghai, China) (protease and phosphatase inhibitors containing). The lysates were subjected to SDS-polyacrylamide gel electrophoresis (SDS-PAGE) (Epizyme, Shanghai, China) and transferred to NC membranes (Amersham, Arlington Heights, IL, USA). The bound antibodies were detected with ECL reagent (Epizyme). β-actin (Sigma) was used as inter control. Images were acquired using a chemiluminescence imaging system (Tanon, Shanghai, China). The intensities of the bands were measured by Image J software. The antibodies used included anti-SLC26A3 (Abcam), anti-CA9 (Abcam), anti-HuR (Protientech, Wuhan, China), anti-OCT4 (Abcam), anti-SOX2 (Abcam), anti-CD133 (Sigma), anti-CD44 (Sigma), anti-pan-Kla (PTO BIO, Hangzhou, China), anti-CD44 (Sigma), anti-Occludin (Protientech), anti-Ecadherin (Abcam).

### Quantitative real-time PCR

Real-time PCR was performed using the 2× Color SYBR Green qPCR Master Mix kit (EZB, San Diego, CA, USA). Total RNA from cells was extracted using the EZ-press RNA Purification kit (EZB). cDNA synthesis was carried out with the Color Reverse Transcription kit (EZB). PCR amplification was conducted using the CFX96 Touch System (BioRad, Hercules, CA, USA). Ct values were normalized to the β-actin gene. The relative expression levels of target genes were determined using the ΔΔCt method. All samples were run in triplicate for each experiment. All primers were synthesized by Tsingke (Beijing, China), and the sequences were listed in Supplementary Table S1.

### Protein stability assay

CHX (MCE, Monmouth Junction, NJ, USA) was prepared in DMSO (Sigma) at a final concentration of 50 mg/mL. CHX was freshly dissolved to ensure maximum activity. 1 μL of CHX was slowly added to 1 mL of cell culture medium and mixed thoroughly. Cells were collected at 0 h, 4 h, 8 h, and 12 h, and proteins were extracted for Western blot analysis to detect the expression of the target protein [48].

### mRNA stability assay

A 1 mg/mL ActD (Sigma) stock solution was prepared in DMSO (Sigma). 30 μL of the ActD stock solution was added to every 100 μL of culture medium and mixed thoroughly. Cells were collected at 0 h, 4 h, 8 h, and 12 h, and RNA was extracted for qPCR analysis to detect the expression of the target gene [49].

### Co-immunoprecipitation

A certain amount of cells was collected and lysed on ice for 20 min in Western and IP lysis (Beyotime) buffer supplemented with PMSF (Beyotime). The lysate was then centrifuged at 12,000 × *g* for 15 min, then aspirate a portion of the supernatant to use as a positive control (input). Magnetic protein A/G beads (MCE) were incubated with antibodies at 4 °C for 3 h, then added to the remaining supernatant and incubated overnight at 4 °C. The beads were washed five times with Western and IP lysis buffer, and proteins were eluted by boiling the beads in 1× loading buffer (Epizyme) at 98°C for 15 min, followed by Western blot.

### RNA immunoprecipitation

A total of 5 × 10^6^ cultured cells were used per test, and 4 μg of anti-HuR antibody (Proteintech, Wuhan, China) or 1 μg of isotype-control antibody (mouse IgG) was used per test. PCR and real-time quantitative PCR (qPCR) were used to verify the binding ability between HuR and the promoters of target genes. The data were calculated as the percentage of the input. Three independent experiments were repeated [50]. The RIP kit was from Sigma.

### Transwell

Matrigel (25 μL) (Corning, N.Y, USA) was added to the upper chamber of transwell inserts (Corning), covering the entire polycarbonate membrane, and was polymerized into a gel at 37 °C for 30 min. Cells were serum-starved by culturing in serum-free DMEM at 37 °C and 5% CO_2_ for 12 h. In a 24-well cell culture plate (Corning), 600 μL of complete DMEM medium containing serum was added to each well. The transwell chambers were then placed into the wells, and 100 μL of serum-free medium containing a cell suspension (1 × 10^5^ cells per well) was added to the upper chamber. The cells were incubated for 24–48 h. The cells were then fixed with 4% PFA (Biosharp), stained with crystal violet (Servicebio). Images acquisition was performed using an Olympus upright microscope and performed cell counting using ImageJ.

### Wound-healing assay

A line was scratched across the central area of a monolayer of adherent cells in an in vitro culture dish using a micropipette tip, removing the cells in the central region. The medium was then replaced with a culture medium containing 1% FBS, and the cells were further cultured. After 24 and 48 h of incubation, the cell culture plates were removed, and the growth of cells around the scratch was observed to determine if they had migrated into the central scratched area. Images and data acquisition were performed using an Olympus upright microscope.

### Cancer stem cells tumor sphere formation

Tumor cells were collected and re-suspended in 1 mL of DMEM/F12 stem medium [11]. The cells were then stained with trypan blue to count viable cells. The cell concentration was set at 1 × 10^4^ cells/mL, and the required volume of the cell suspension was calculated. Six replicate wells were set up. The cells were seeded into a 96-well plate at 100 μL per well and cultured at 37 °C with 5% CO_2_. The number of tumor spheres with a diameter greater than 50 μM was counted over a period of 1–2 weeks [51]. Images and data acquisition were performed using an Olympus upright microscope.

### Limiting dilution analysis

Tumor cells were collected and re-suspended in 1 mL of DMEM/F12 stem medium. The cells were then stained with trypan blue to count viable cells. A cell concentration gradient was set up at 125, 62, 31, 15, 8, 4, and 2 cells per well, with six replicate wells for each concentration. The required volume of the cell suspension was calculated, and the cells were seeded into a 96-well plate at 100 μL per well. The cells were cultured at 37 °C with 5% CO_2_. After 7–10 days, the proportion of wells without tumor spheres was calculated [51]. Images and data acquisition were performed using an Olympus upright microscope.

### OXA resistance assay

Tumor cells were collected and re-suspended in 1 mL of medium. The cells were stained with trypan blue to count viable cells and seeded into a 96-well plate at ~4000 cells per well, with 100 μL of medium added to each well. OXA (Sigma) was added to the medium with a set concentration gradient. After 48 h of incubation, 20 μL of MTS (Sigma) was added to each well, and the cells were incubated at 5% CO_2_ for 1–4 h. Data were then read at an absorbance peak of 490 nm by microplate reader (Thermo).

### Statistic analysis

Both in-vitro and in-vivo experiments employed simple randomisation. All in vitro experiments were conducted as three independent experiments, each with separate treatment and measurement. The sample size was determined based on previously published study and our own pilot experiments. Data were expressed as the means ± SDs and were analyzed by SPSS14.0 software (SPSS, IL). No significant difference in variance between groups. Student’s *t* test was used for statistical analysis (two-sided test), and a *P* < 0.05 was considered statistically significant.

### Animal studies

Subcutaneous xenograft models were established as described previously [33]. We estimated that five mice per group would provide ≧80% power (α = 0.05) to detect a biologically relevant difference. Briefly, 1 × 10^4^ DLD1 cells were injected subcutaneously into male 4–6 week old BALB/c-nu mice (Weitong Lihua, Beijing, China) in 0.1 mL of matrigel and PBS (1:4). After 1 week, 100 ml 200 mM NaHCO_3_ or 10 mg/kg OXA was provided to the mice and remained available two or three times a week [28]. The tumor volumes of the mice in each group (*n* = 5) were estimated each week using the formula V = ab^2^/2 (v, volume; a, length; b, width). After 22 days, the tumor tissues were dissected, and the weights were measured.

For the in vivo therapeutic study of AZD3965, tumors derived from the same colorectal cancer patient were xenografted subcutaneously into male 4–6 week old BALB/c-nu mice. After 7 days, the mice were randomly divided into treatment and control groups (*n* = 5). The treatment group received AZD3965 at 50 mg/kg orally once daily; the control group received an equal volume of saline. After 14 days of treatment, administration was stopped, and the animals were further monitored for 7 days to assess tumor growth [52].

During the in-vivo efficacy study, the investigator who performed daily tumor-size measurements and endpoint assessments was blinded to group allocation. Treatment codes were revealed only after all caliper readings and statistical analyses had been completed. Blinding was maintained by having an independent technician prepare and label all drug solutions and by using coded ear tags. Inclusion/exclusion criteria were pre-established. Inclusion: tumors reaching 50–150 mm³within 10 days post-injection; animals with body-weight loss ≦10%. Exclusion: ulcerated or necrotic tumors; weight loss >10%; infection or unrelated morbidity. No animals/tumors were excluded from the final analysis.

## Supplementary information

Supplementary

Uncropped western blots

Original data

Original data

## Data Availability

The datasets generated during and/or analyzed during the current study are available from the corresponding author on reasonable request.
