# Supplementary material for: Lactylation of SLC26A3 in the acidic tumor microenvironment promotes malignant progression of colorectal carcinoma
Source: Cell Death Dis. 2026 Jan 30;17(1):164. doi: 10.1038/s41419-026-08422-9 (PMC12877128; doi:10.1038/s41419-026-08422-9)
Supplement: Supplementary file 1 — Supplementary [file 41419_2026_8422_MOESM1_ESM.doc]

**Supplemental Materials to:**

**Lactylation of SLC26A3 in the Acidic Tumor Micro****environment Promotes Malignant Progression of Colorectal Carcinoma**

Chong Chen^1,2,3^*^#^*, Du Cai^1,2,3^*^#^*, Xuanhui Liu^1,2,3^*^#^*, Yifan Zheng^1,2,3^, Xinxin Huang^1,2,3^, Dongwen Chen^1,2,3^, Jiawei Cai^1,2,3^,Yiran Bie^1,2,3^, Zhengran Zhou^1,2,3^, Chuling Hu^1,2,3^, Zhengyu Wei^1,2,3^, Kuntai Cai^7^, Ting Li^4^, Shuzhen Luo^5,6^, Dongbing Liu^5,6^, Kui Wu^5,6^, Zerong Cai^1,2,3^, Feng Gao^1,2,3^, Xiaojian Wu^1,2,3*^, Peishan Hu^1,2,3*^

^1^Department of General Surgery (Colorectal Surgery), The Sixth Affiliated Hospital, Sun Yat-sen University, Guangzhou, China

^2^Guangdong Provincial Key Laboratory of Colorectal and Pelvic Floor Diseases, The Sixth Affiliated Hospital, Sun Yat-Sen University, Guangzhou, China.

^3^Biomedical Innovation Center, The Sixth Affiliated Hospital, Sun Yat-sen University, Guangzhou, China

^4^Department of Gastroenterology and Urology, The Affiliated Cancer Hospital of Xiangya School of Medicine, Central South University/Hunan Cancer Hospital, Changsha 410013, P. R. China.

^5^Institute of Intelligent Medical Research (IIMR), BGI Genomics, Shenzhen, China.

^6^Guangdong Provincial Key Laboratory of Human Disease Genomics, BGI Research, Shenzhen, China.

^7^Precision Medicine Center, Affiliated Hospital of Guangdong Medical University, Zhanjiang, Guangdong 524001, P.R. China.

*#* These authors have equal contributions

* Corresponding Author

Xiaojian Wu Ph.D. E-mail: [wuxjian@mail.sysu.edu.cn;](mailto:wuxjian@mail.sysu.edu.cn;)

Peishan Hu Ph.D. E-mail: [hupsh3@mail.sysu.edu.cn](mailto:hupsh3@mail.sysu.edu.cn)

The authors declare no competing financial interests.

**Files in this data supplement:**

**Supplementary figures and figure legends**

**Supplementary tables S1**

**Figure S1**

**
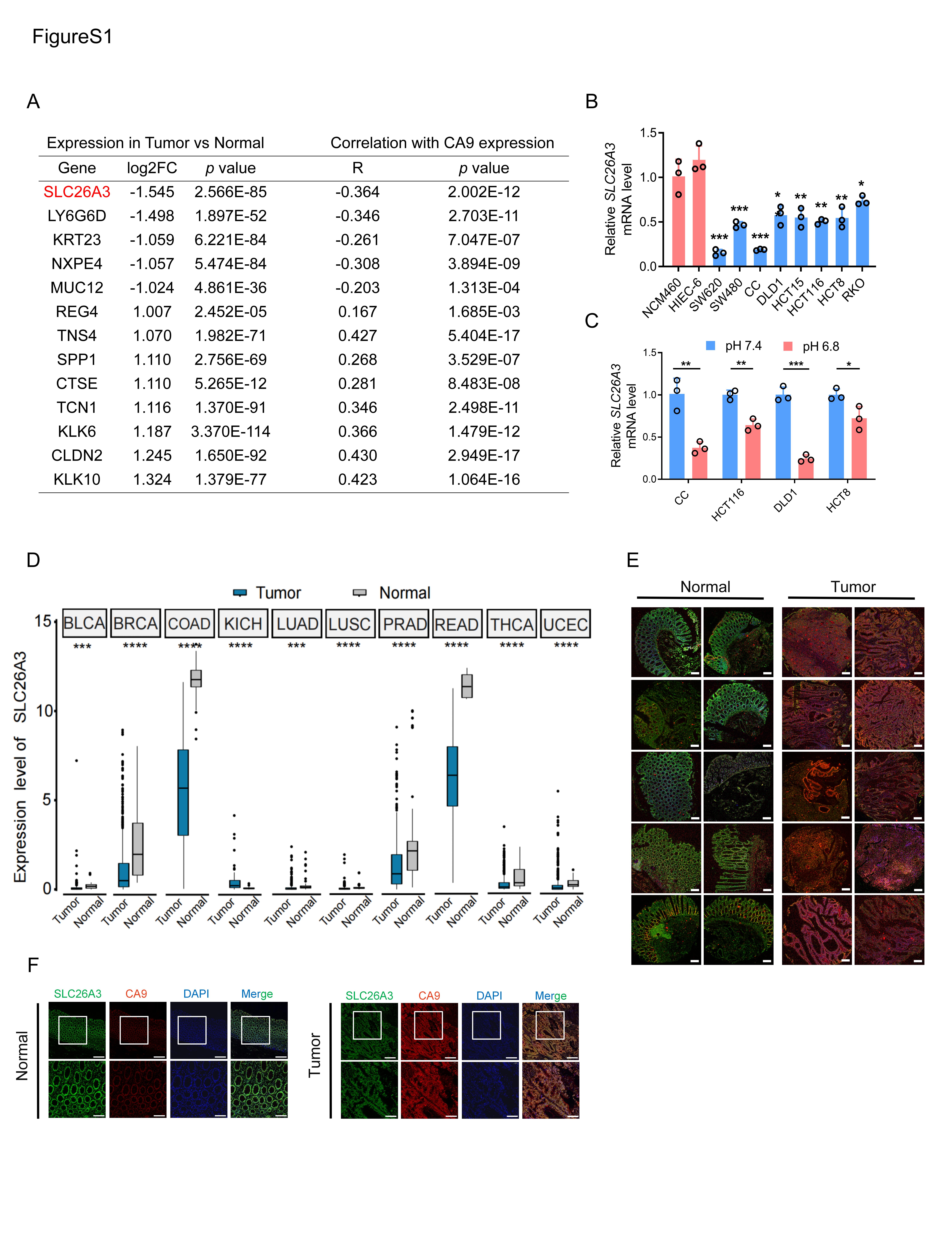
**

**Figure S1. SLC26A3 expression is negatively correlated with acidic microenvironment associated CRC malignanc. A** Genes related to CA9 expression in the COCC database and their expression levels in cancer and adjacent non-cancerous tissues. **B** qPCR of SLC26A3 expression in normal intestinal epithelial cells and CRC cell lines. Student’s t-test. **C** qPCR of SLC26A3 expression under pH 7.4/6.8 culture conditions. Student’s t-test. **D** Expression of SLC26A3 in cancer and adjacent non-cancerous tissues across multiple cancer types. **E** Multiplex immunofluorescence staining of SLC26A3 and CA9 in tissue chips of CRC patients. Scale bars: 200μm. **F** Immunofluorescence of SLC26A3 and CA9 in tumor and normal tissue of CRC patient. Scale bars: 100μm. *P < 0.05, **P < 0.01, and ***P < 0.001,Data are representative of three independent experiments

**Figure S2**


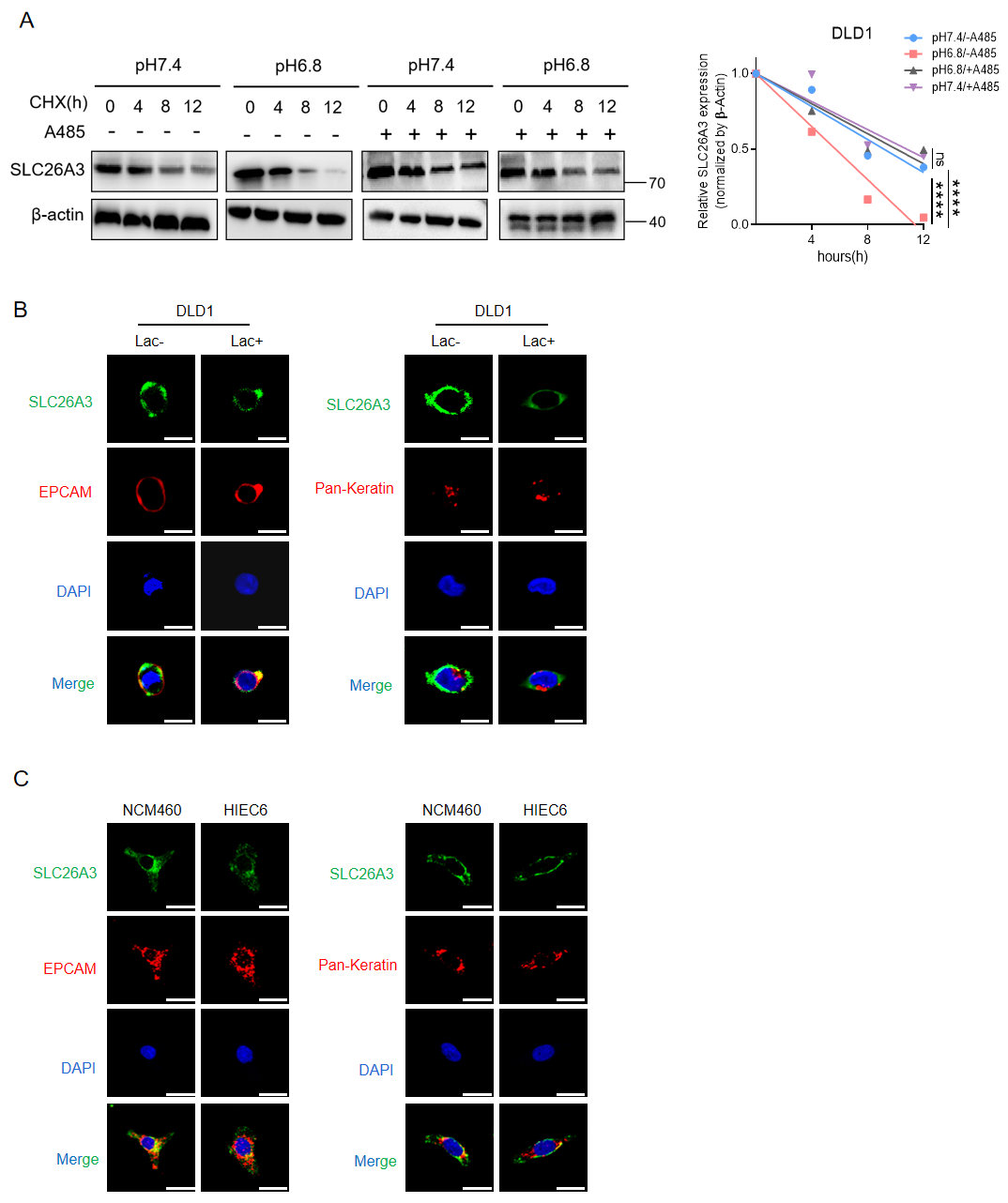


**Figure S2. Lactylation of SLC26A3 in acidic environment reduces its stability and expression.**

**A** Immunoblotting to detect SLC26A3 expression under pH7.4/6.8, and A485 or non-A485 treatment. Quantified immunoblotting results using ImageJ. Two-way Anova. **B** Co-localization of SLC26A3 with membrane markers(EPCAM) and cytosolic markers(Pan-Keratin) in DLD1 cells. Scale bars: 20μm. **C** Co-localization of SLC26A3 with membrane markers(EPCAM) and cytosolic markers(Pan-Keratin) in NCM460 and HIEC6 cells. Scale bars: 20μm. *P < 0.05, **P < 0.01, and ***P < 0.001, Data are representative of three independent experiments

**Figure S3**


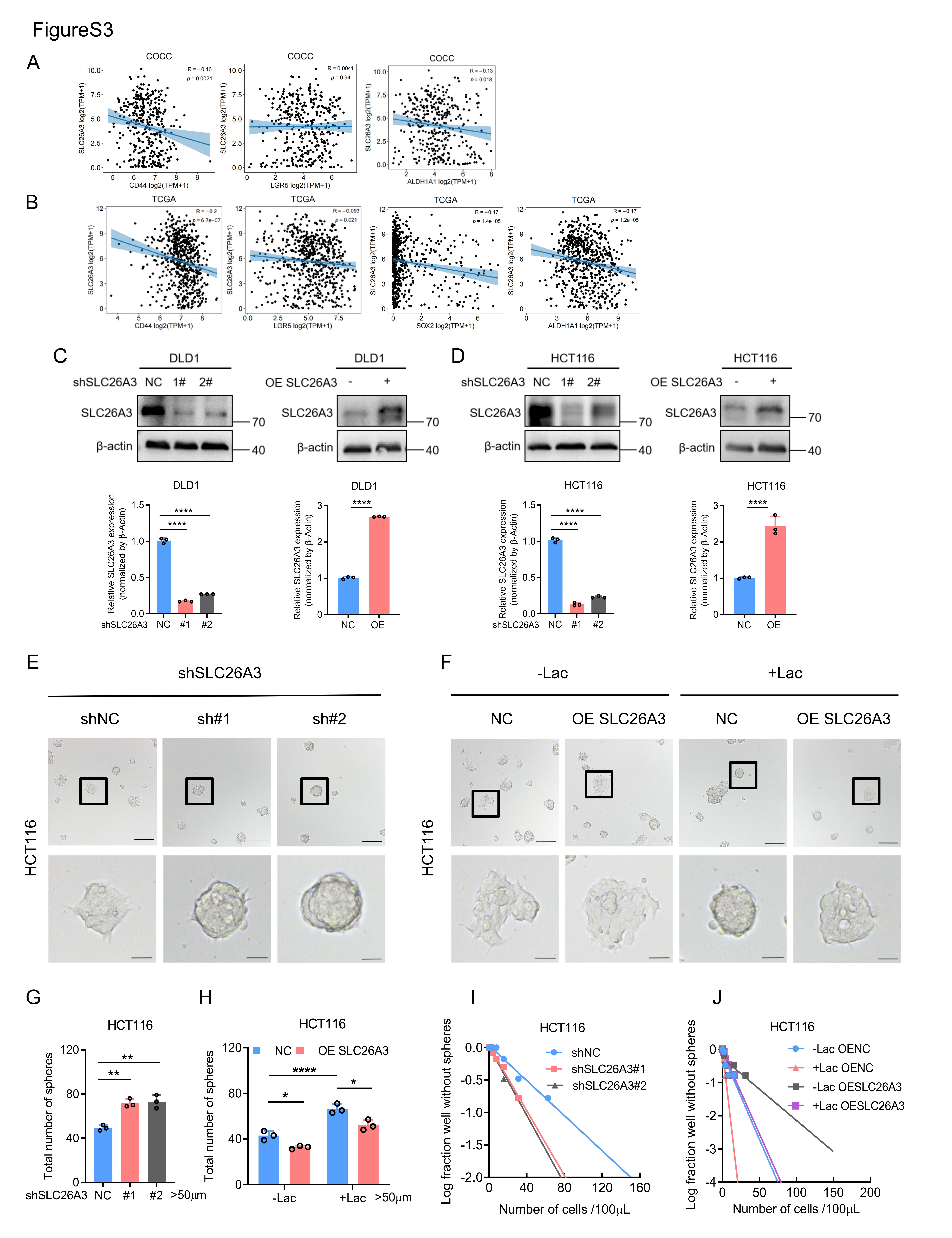


**Figure S3. Low SLC26A3 levels drive stemness of tumor cells.** **A-B** Correlation between the expression of SLC26A3 and stemness markers in the COCC and TCGA databases. Pearson correlation analysis. **C-D** Immunoblotting and quantification of DLD1 and HCT116 cells transfected with lentivirus to knock down or overexpress SLC26A3. Quantified immunoblotting results using ImageJ. Student’s t-test. **E,G** Representative images (E) and quantified data (G) for tumor spheres (with diameters larger than 50 µm) formed by control and SLC26A3-targeting shRNA treated HCT116 cells. Scale bars: upper, 200μm; lower, 50μm. Student’s t-test. **F,H** Representative images (F) and quantified data (H) for tumor spheres (with diameters larger than 50µm) formed by control and SLC26A3-overexpress HCT116 cells. Scale bars: upper, 200μm; lower, 50μm. Student’s t-test. **I-J** Limiting dilution assay of HCT116 cells treated with SLC26A3-targeting shRNA(I) or SLC26A3-overexpress(J).*P < 0.05, **P < 0.01, and ***P < 0.001, Data are representative of three independent experiments

**FigureS4
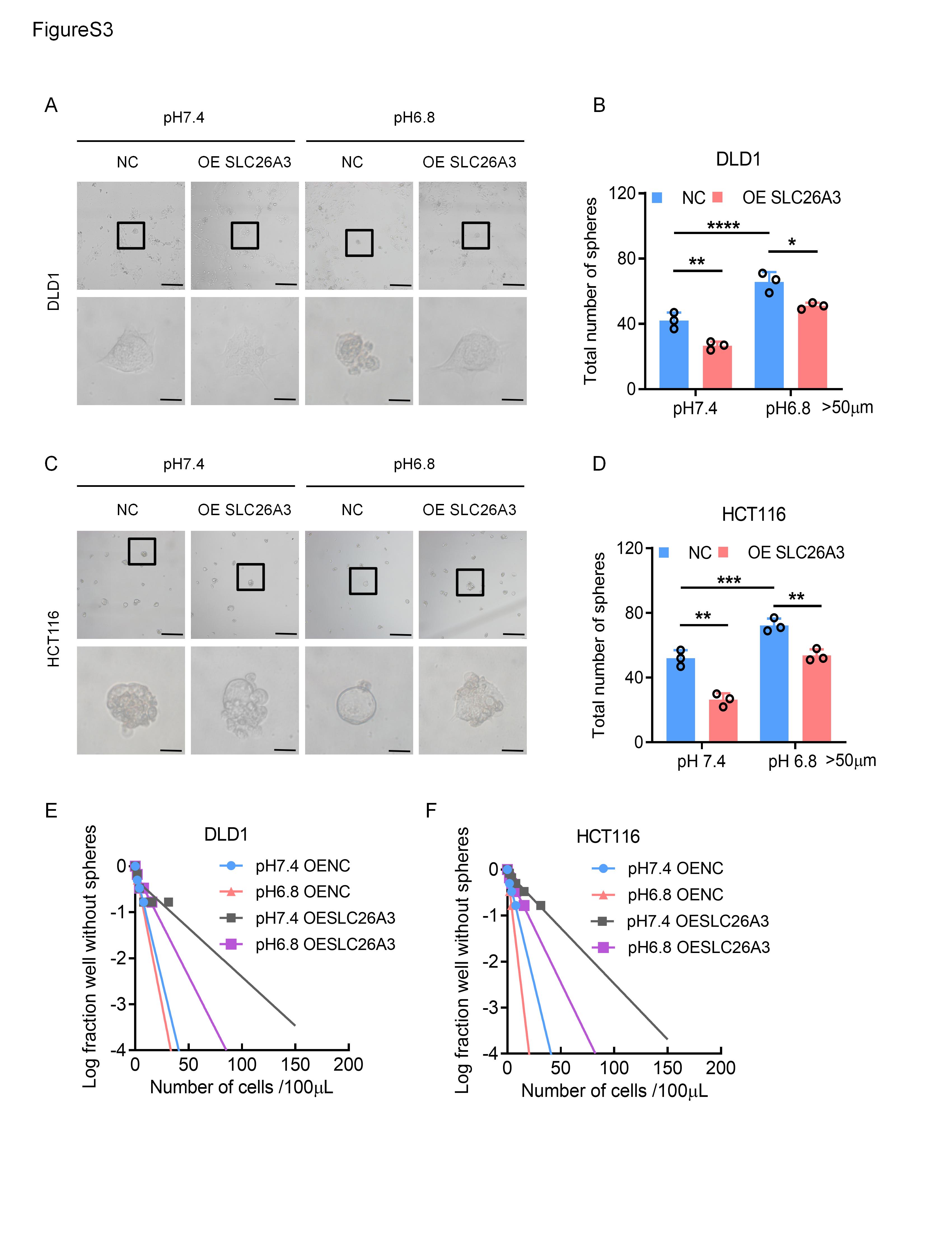
**

**Figure S4. Low SLC26A3 levels drive stemness of tumor cells.** **A-D** Representative images (A,C) and quantified data (B,D) for tumor spheres (with diameters larger than 50µm) formed by DLD1 and HCT116 cells which were cultured at pH 7.4 or 6.8. Scale bars: upper, 200μm; lower, 50μm. Student’s t-test. **E-F** Limiting dilution assay of DLD1 and HCT116 cells which were cultured at pH 7.4 or 6.8. *P < 0.05, **P < 0.01, and ***P < 0.001, Data are representative of three independent experiments

**Figure S5**

**
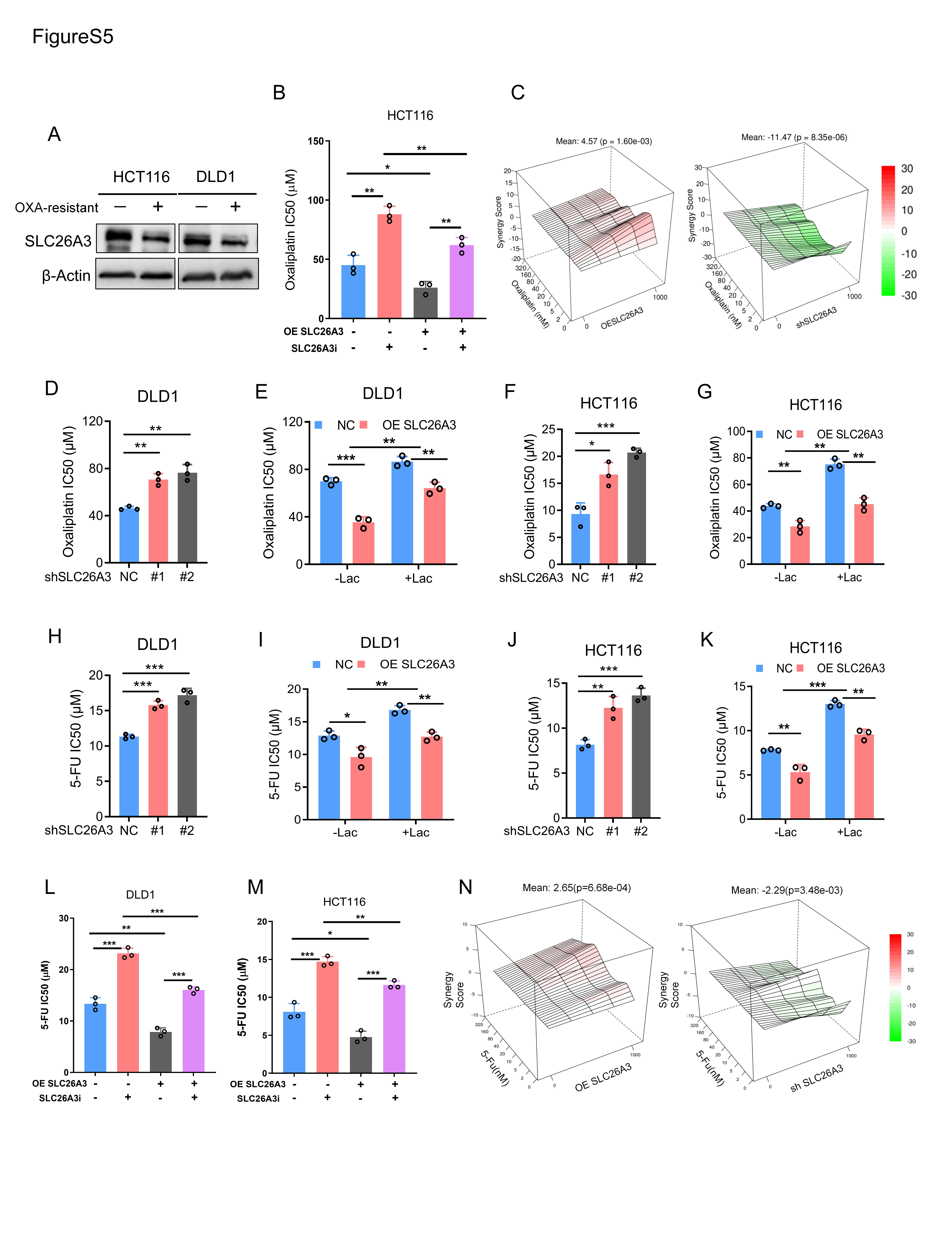
**

**Figure S5. Low SLC26A3 expression leads to chemotherapy resistance of CRC Cells.**

**A** Immunoblotting of SLC26A3 expression in OXA-resistant HCT116 and DLD1 cells. **B** Cell viability of control and SLC26A3-overexpress HCT116 cells with SLC26A3 inhibitor. Student’s t-test. **C** Bliss synergistic analysis shows that overexpression of SLC26A3 has synergistic effect with OXA, and knockdown SLC26A3 has antagonistic effect with OXA in HCT116 cells. **D-G** Cell viability of DLD1 and HCT116 cells with OXA and lactate treatment. Student’s t-test. **H-K** Cell viability of DLD1 and HCT116 cells with 5-FU and lactate treatment. Student’s t-test. **L-M** Cell viability of control and SLC26A3-overexpress HCT116/DLD1 cells with SLC26A3 inhibitor. Student’s t-test. **N** Bliss synergistic analysis shows that overexpression of SLC26A3 has synergistic effect with 5-FU, and knockdown SLC26A3 has antagonistic effect with OXA in HCT116 cells.*P < 0.05, **P < 0.01, and ***P < 0.001, Data are representative of three independent experiments

**Figure S6**


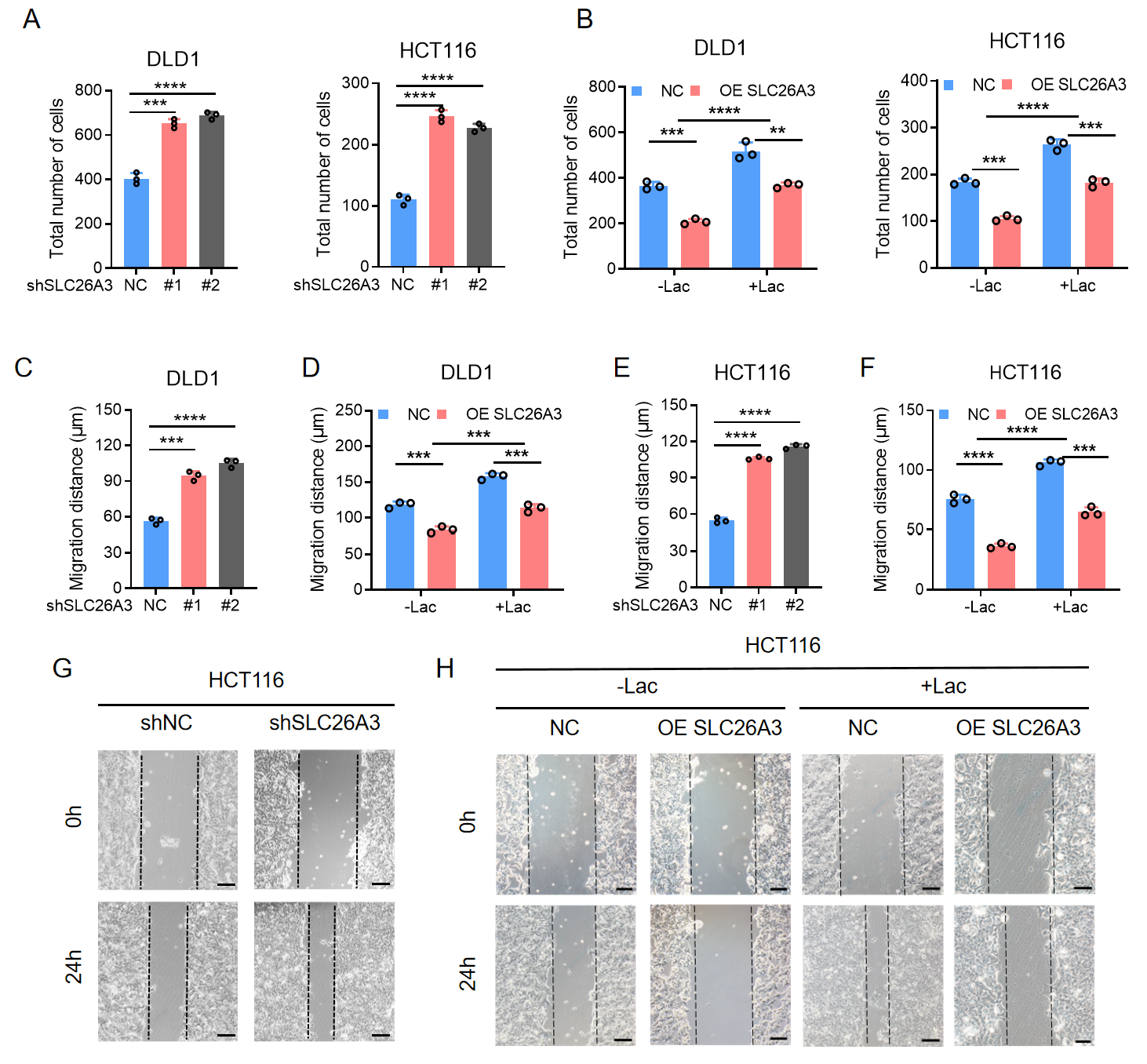


**Figure S6. Low SLC26A3 expression enhances invasion and migration of CRC Cells.** **A-B** Quantified data of the transwell assay. Student’s t-test. **C-F** Quantified data of the wound-healing assay. Student’s t-test. **G-H** Representative images of the wound-healing assay. Scale bars: 50μm. *P < 0.05, **P < 0.01, and ***P < 0.001, Data are representative of three independent experiments

**Figure S7**


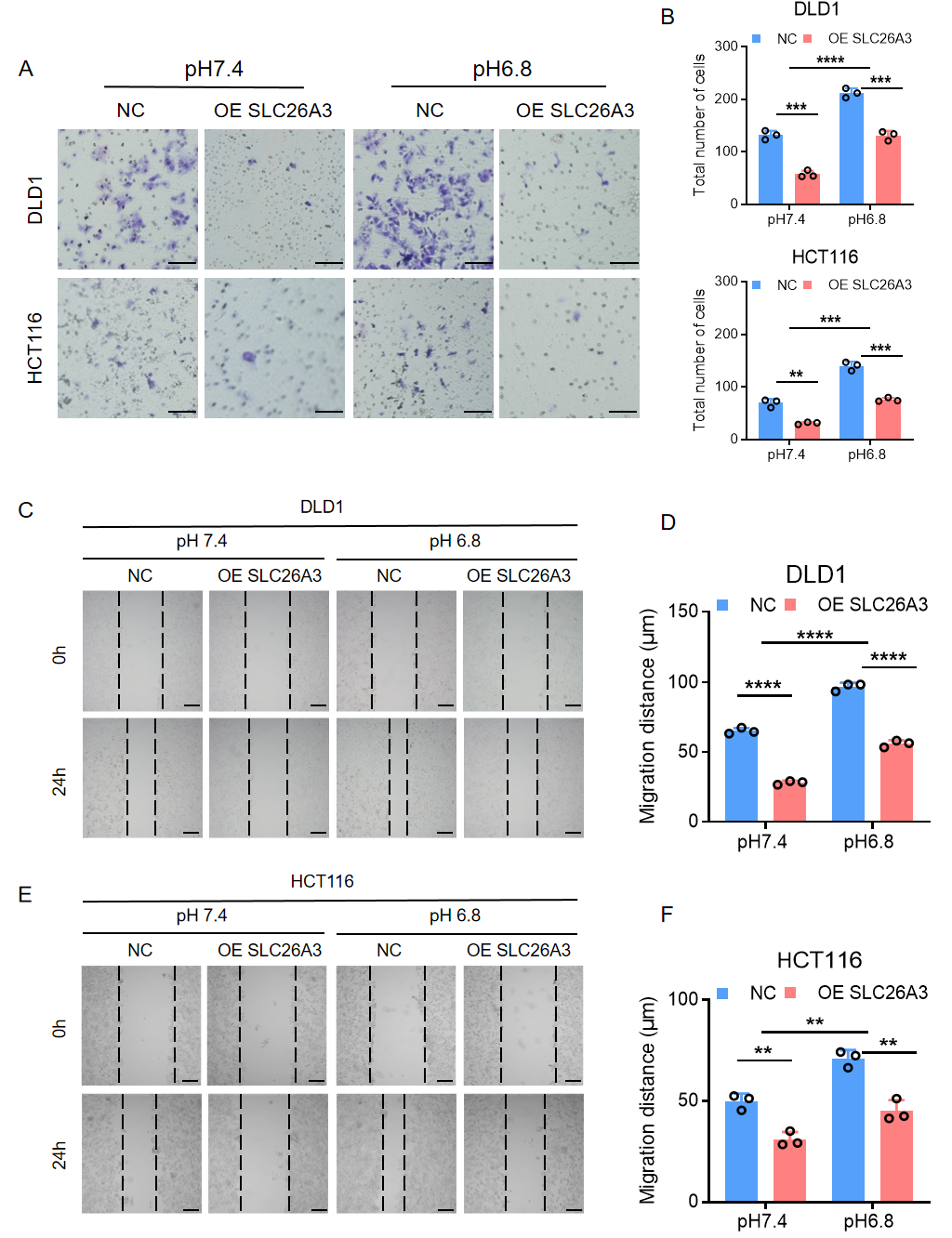


**Figure S7. Low SLC26A3 expression leads to chemotherapy resistance and enhances invasion and migration of CRC Cells.** **A-B** Representative images and quantified data of the transwell assay in pH 7.4 or 6.8 environment. Scale bars: 100μm. Student’s t-test. **C-F** Representative images and quantified data of the wound-healing assay in pH 7.4 or 6.8 environment. Scale bars: 50μm. Student’s t-test. *P < 0.05, **P < 0.01, and ***P < 0.001, Data are representative of three independent experiments

**Figure S8**


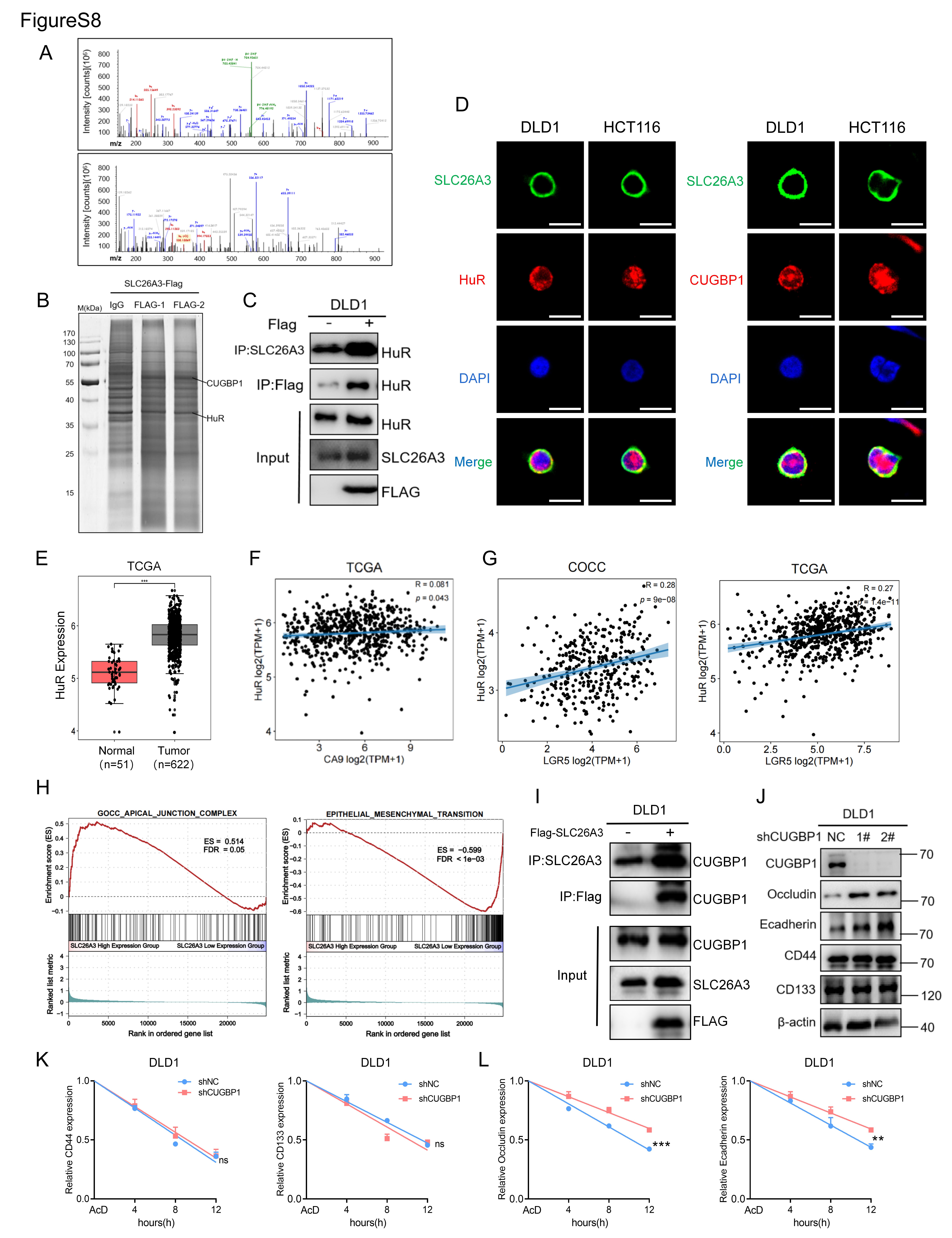


**Figure S8. SLC26A3 interacts with HuR/CUGBP1 and regulates the expression of genes related to malignant phenotype of tumor cells. A-B** Immunoprecipitation experiments (B) and mass spectrometry results (A) indicate that SLC26A3 interacts with HuR/CUGBP1. **C** co-IP assay shows that there is protein interaction between SLC26A3 and HuR. **D** Immunofluorescence revealed that SLC26A3 co-localizes with both HuR and CUGBP1. Scale bars: 20μm. **E** Analysis of the TCGA database indicates that HuR is highly expressed in CRC. Wilcox rank sum test. **F** Analysis of the TCGA database indicates that HuR expression is positively correlated with CA9 expression in CRC. Pearson correlation analysis. **G** Analysis of the COCC and TCGA database indicates that HuR expression is positively correlated with expression of stemness mark LGR5 in CRC. Pearson correlation analysis. **H** Pathway-based GSEA revealed that SLC26A3 is related to the expression of epithelial junction proteins and EMT. **I** co-IP assay shows that there is protein interaction between SLC26A3 and CUGBP1. **J** Immunoblotting of related marker after knockdown of CUGBP1 in DLD1 cells. **K-L** qPCR of related mRNA after ActD treatment in control and CUGBP1 knock-down group in DLD1 cells. Two-way anova test. *P < 0.05, **P < 0.01, and ***P < 0.001, Data are representative of three independent experiments

**Figure S9**


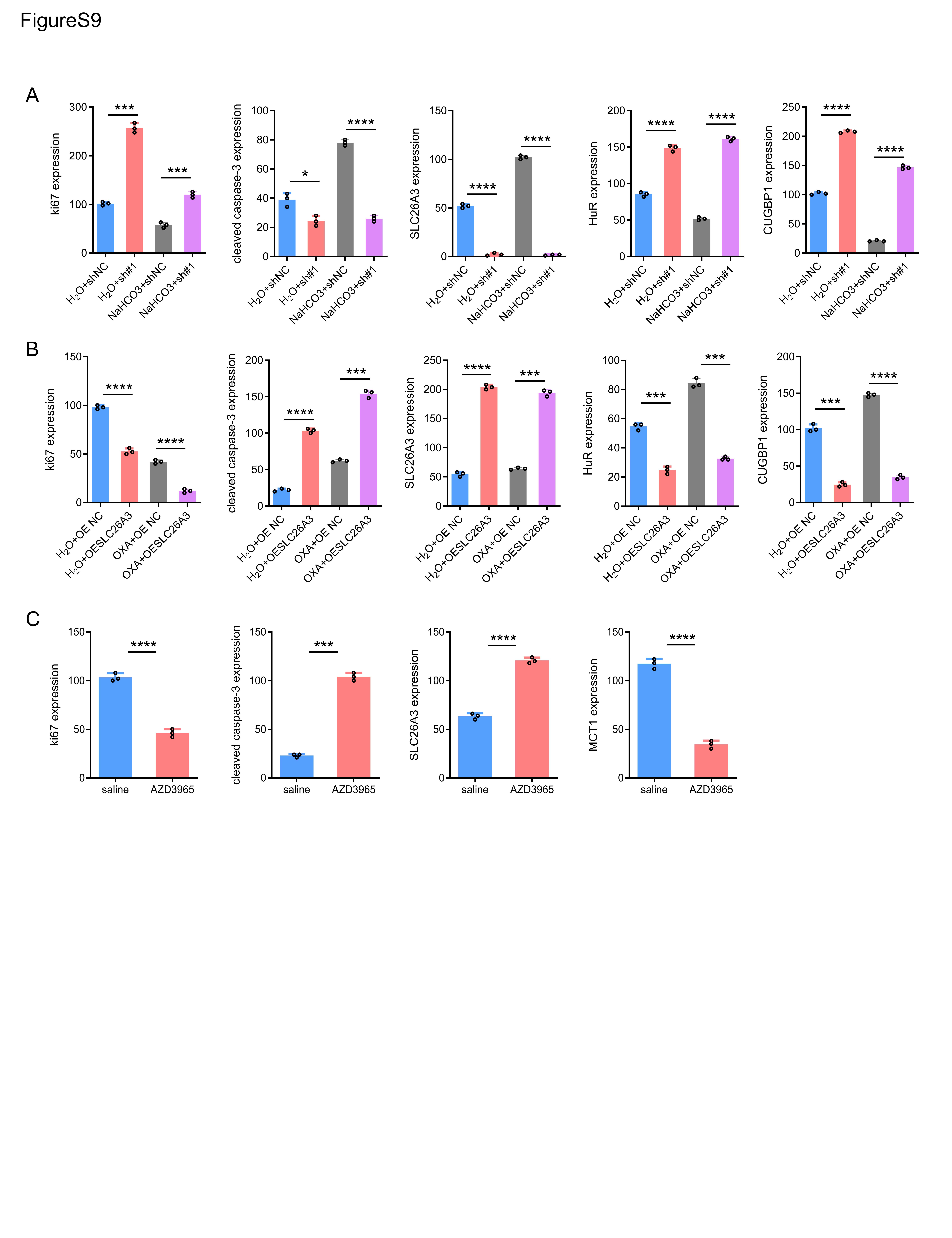
**Figure S9. A-C** H-Score of the IHC experiment in Fig.7J-L. Student’s t-test. *P < 0.05, **P < 0.01, and ***P < 0.001, Data are representative of three independent experiments

**Supplementary Table S1: Oligonucleotides used for plasmid construction, real time PCR and RIP experiments.**

| **Plasmid Constructs (Human)** | |  |
| --- | --- | --- |
| SLC26A3 | Forward | 5´-GCTCAAGCTTCGAATTCGCCACCATGATTGAACCCTTTGGGAATCAGTATATTG-3´ |
|  | Reverse | 5´-TAGAGTCGCGGGATCCTTATTTGTC-3´ |
| **Quatitative real time PCR (Human)** | |  |
| SLC26A3 | Forward | 5´-TGCCAGCATACCGGCTTAAAG-3´ |
|  | Reverse | 5´-TCTGGAAGTGCCGAAGAAAAG-3´ |
| CD44 | Forward | 5´-CACACCCTCCCCTCATTCAC-3´ |
|  | Reverse | 5´-TGGATGGCTGGTATGAGCTG-3´ |
| CD133 | Forward | 5´-TGAACTGAGGCAGCTTCCACCC-3´ |
|  | Reverse | 5´-CGACAGTCGTGGTTTGGCGTT-´3 |
| Sox2 | Forward | 5´-TCAGGAGTTGTCAAGGCAGAGAAGA-3´ |
|  | Reverse | 5´-TGCCGCCGCCGATGATTGT-3´ |
| OCT4 | Forward | 5´-TTGGGCTCGAGAAGGATGTGGT-3´ |
|  | Reverse | 5´-ACGGAGACAGGGGGAAAGGCTT-3´ |
| ZO-1 | Forward | 5´-ACCAGTAAGTCGTCCTGATCC-3´ |
|  | Reverse | 5´-TCGGCCAAATCTTCTCACTCC-3´ |
| Occludin | Forward | 5´-ACAAGCGGTTTTATCCAGAGTC-3´ |
|  | Reverse | 5´-GTCATCCACAGGCGAAGTTAAT-3´ |
| Claudin-1 | Forward | 5´-CCTCCTGGGAGTGATAGCAAT-3´ |
|  | Reverse | 5´-GGCAACTAAAATAGCCAGACCT-3´ |
| E-cadherin | Forward | 5´-ATTTTTCCCTCGACACCCGAT-3´ |
|  | Reverse | 5´-TCCCAGGCGTAGACCAAGA-3´ |
| HuR | Forward | 5´-GGCGCAGAGATTCAGGTTCT-3´ |
|  | Reverse | 5´-TGGTCACAAAGCCAAACCCT-3´ |
| β-actin | Forward | 5´-CATGTACGTTGCTATCCAGGC-3´ |
|  | Reverse | 5´-CTCCTTAATGTCACGCACGAT-3´ |
| **RIP (human)** | |  |
| CD44-1 | Forward | 5´-GGACAAGTTTTGGTGGCACG-3´ |
|  | Reverse | 5´-TCCGTCCGAGAGATGCTGTAC-3´ |
| CD44-2 | Forward | 5´-GGGAGTCAAGAAGGTGGAGC-3´ |
|  | Reverse | 5´-GCCAAGAGGGATGCCAAGAT-3´ |
| CD133-1 | Forward | 5´-TGCCTTGAGTGAATGACCCC-3´ |
|  | Reverse | 5´-TTCTGTCTGAGGCTGGCTTG-3´ |
| CD133-2 | Forward | 5´-GCTTACTGTGTGGCGTGTG-3´ |
|  | Reverse | 5´-TCCAACCATGAGGAAGACGC-3´ |
| Occludin-1 | Forward | 5´-GGCGAGCGGATTGGTTTATC-3´ |
|  | Reverse | 5´-GCCTGGATGACATGGCTGAT-3´ |
| Occludin-2 | Forward | 5´- ACTTCAGGCAGCCTCGTTAC-3´ |
|  | Reverse | 5´-CCTGATCCAGTCCTCCTCCA-3´ |
| Ecadherin-1 | Forward | 5´-GTCAGTTCAGACTCCAGCCC-3´ |
|  | Reverse | 5´-GCAGAGCCAAGAGGAGACC-3´ |
| Ecadherin-1 | Forward | 5´-TGAGCTCCCTGAACTCCTCA-3´ |
|  | Reverse | 5´-TCTTGAAGCGATTGCCCCAT-3´ |
| **Knochdown shRNA** | | Target sequence |
| Human  SLC26A3 1# |  | 5´-GACAACAATCAGATAGAATTCAAGAGATTCTATCTGATTGTTGTCC-3´ |
| Human  SLC26A3 2# |  | 5´-GAACCAACATCTATAAGAATTCAAGAGATTCTTATAGATGTTGGTTC-3´ |
| Human HuR 1# |  | 5´-CGTGGATCAGACTACAGGTTT-3´ |
| Human HuR 2# |  | 5´-GCAGCATTGGTGAAGTTGAAT-3´ |
