# Supplementary material for: Lactylation of SLC26A3 in the acidic tumor microenvironment promotes malignant progression of colorectal carcinoma
Source: Cell Death Dis. 2026 Jan 30;17(1):164. doi: 10.1038/s41419-026-08422-9 (PMC12877128; doi:10.1038/s41419-026-08422-9)

Figure1 D

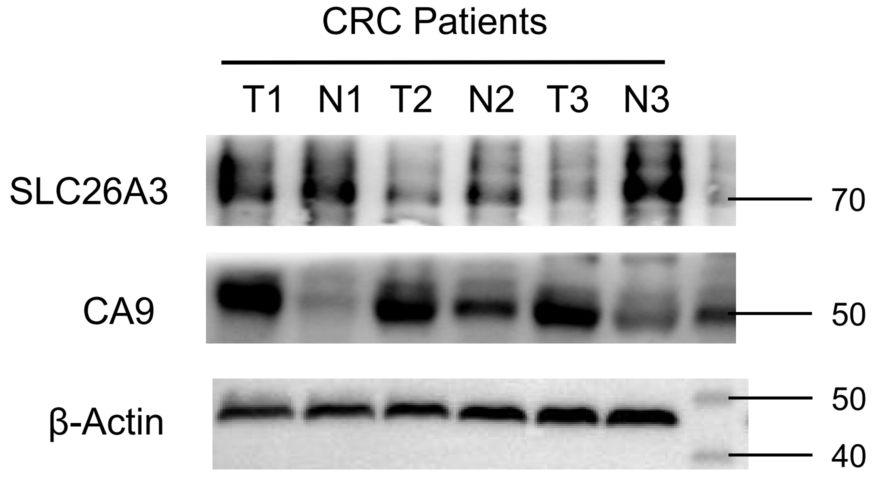

Figure1 E

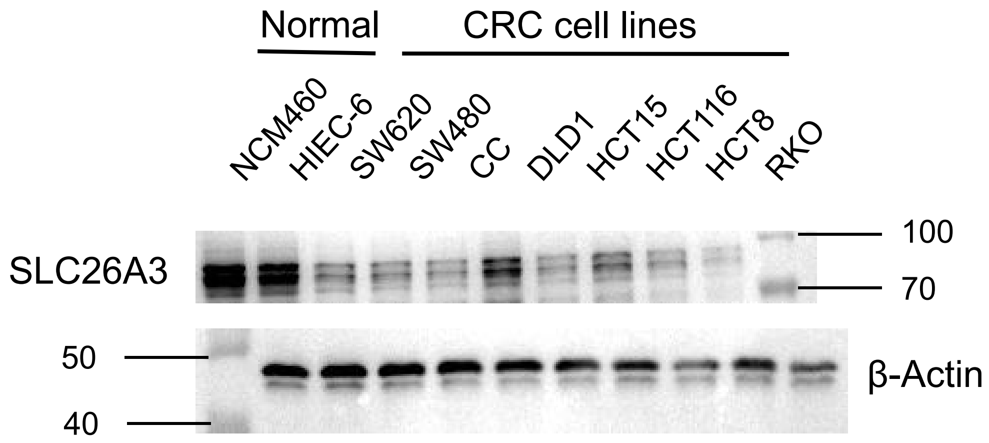

Figure1 J

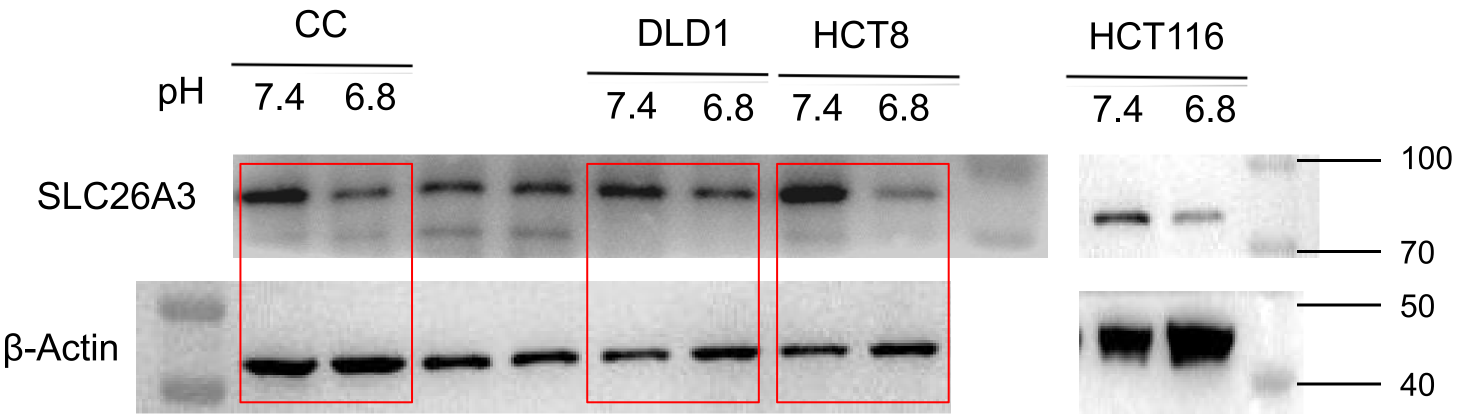

Figure3 B

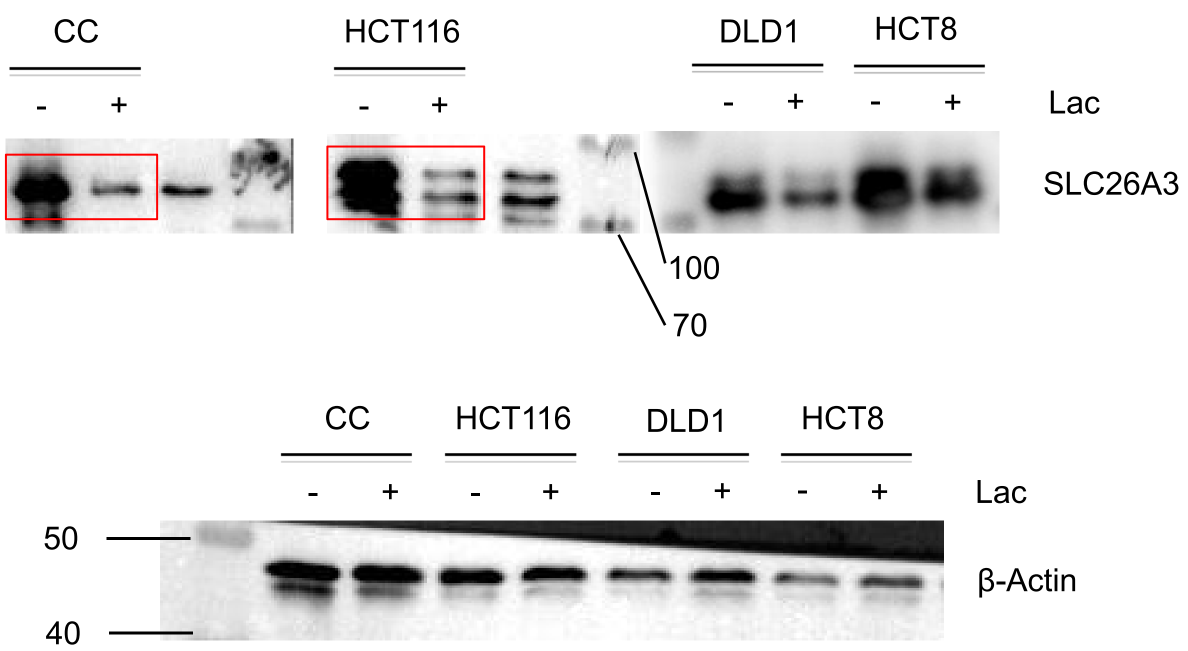

Figure3 D

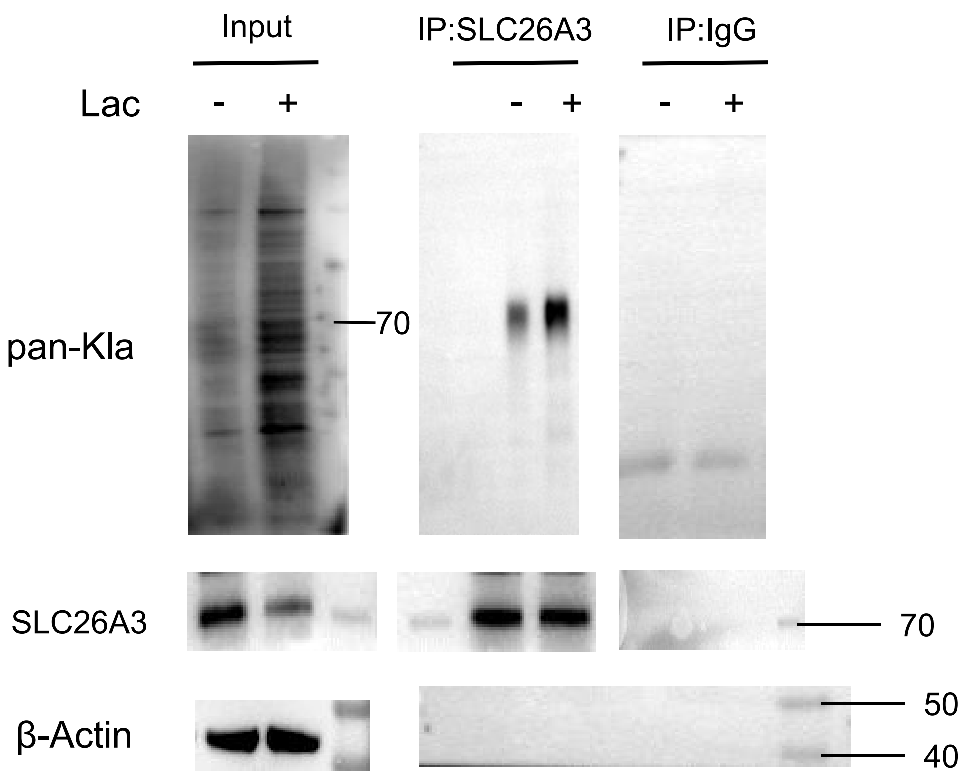

Figure3 F

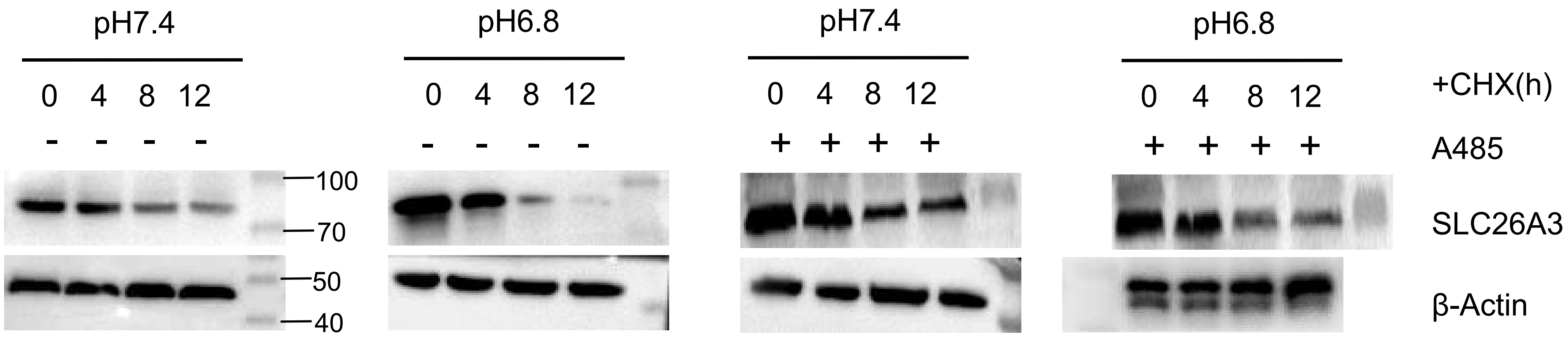

Figure3 H

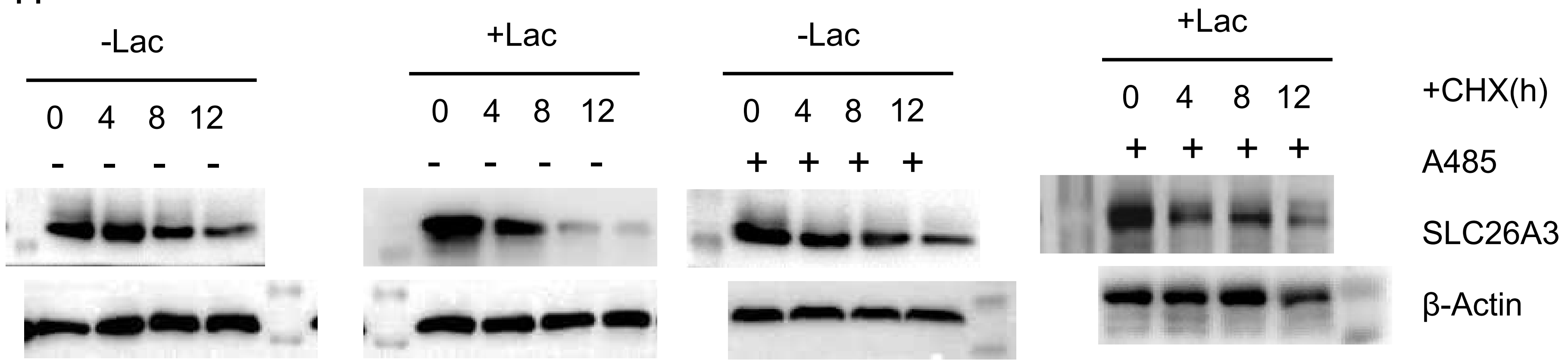

Figure4 A

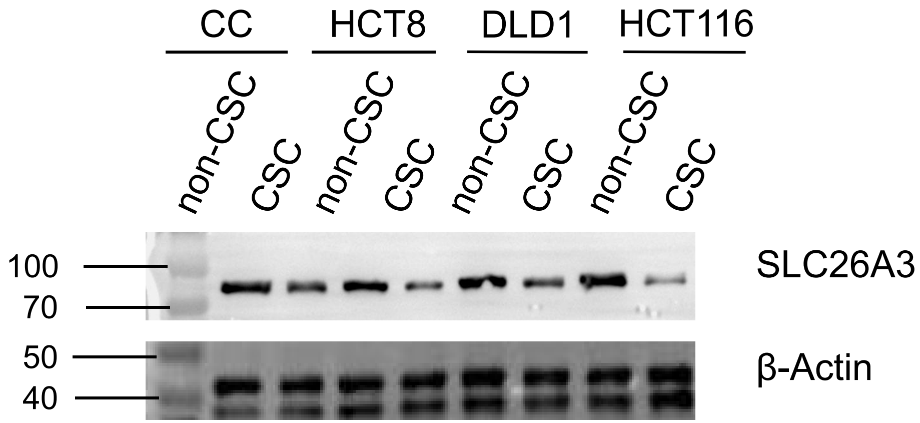

Figure4 D

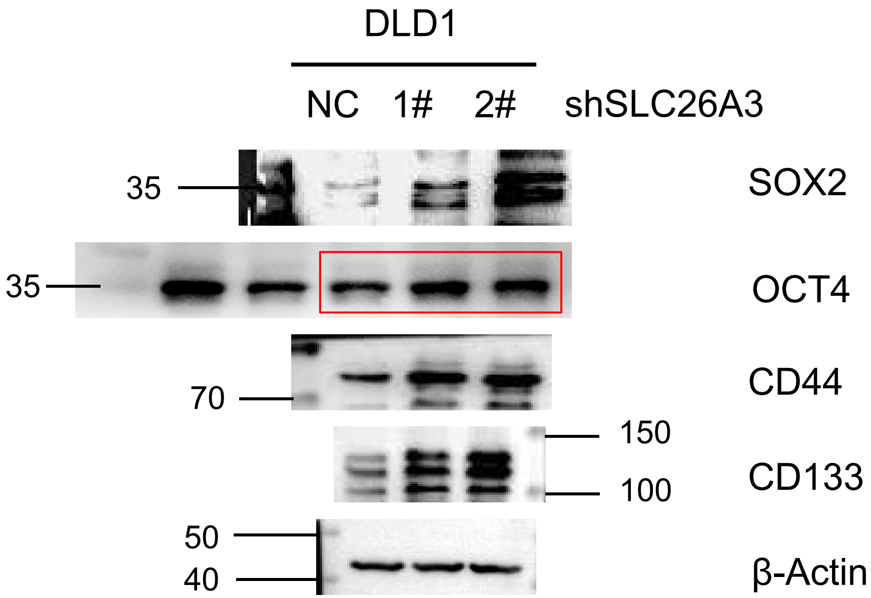

Figure4 D

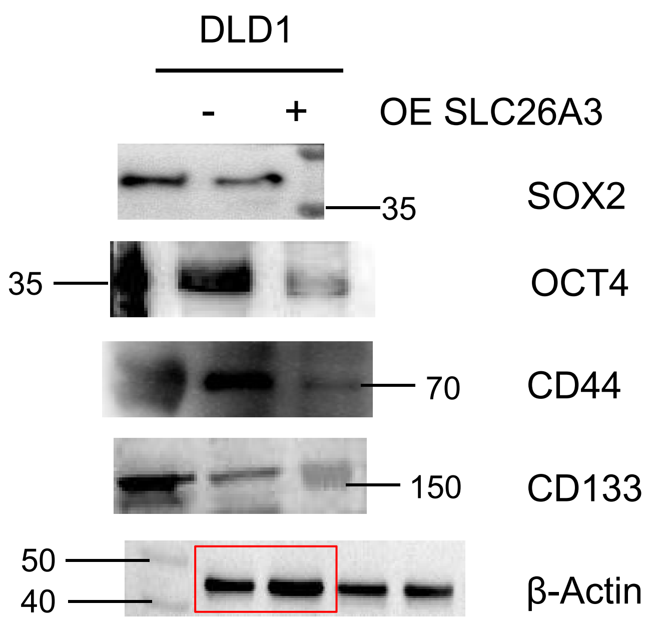

Figure4 E

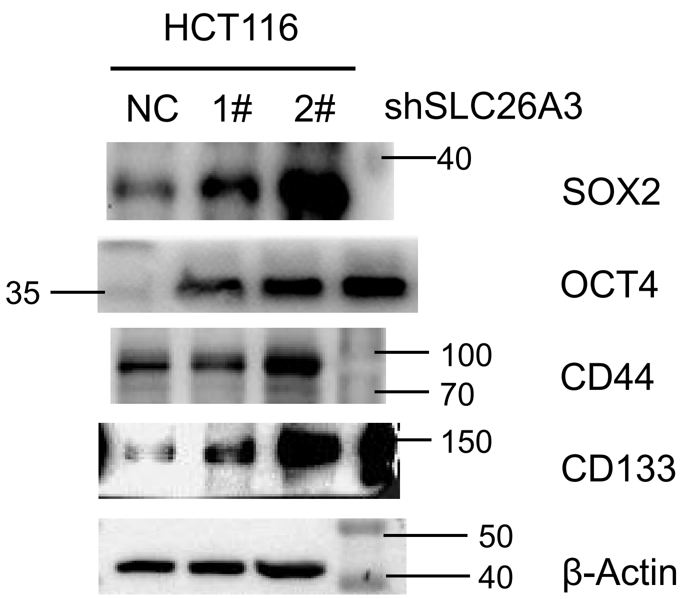

Figure4 E

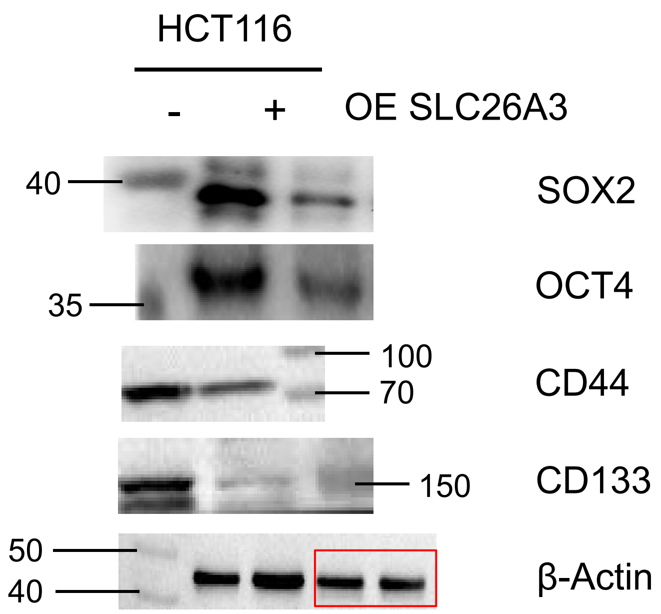

FigureS2 C

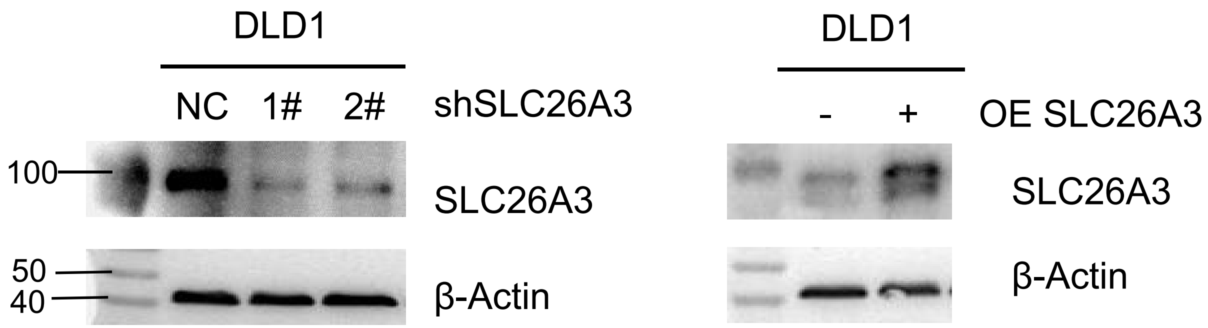

FigureS2 D

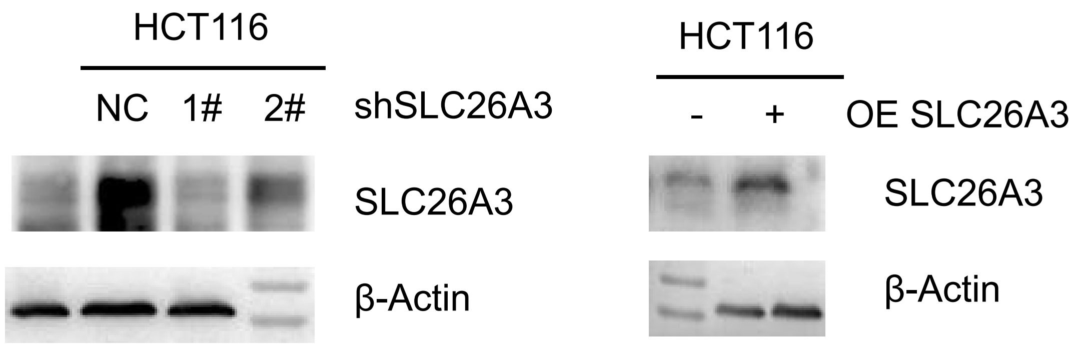

FigureS4 A

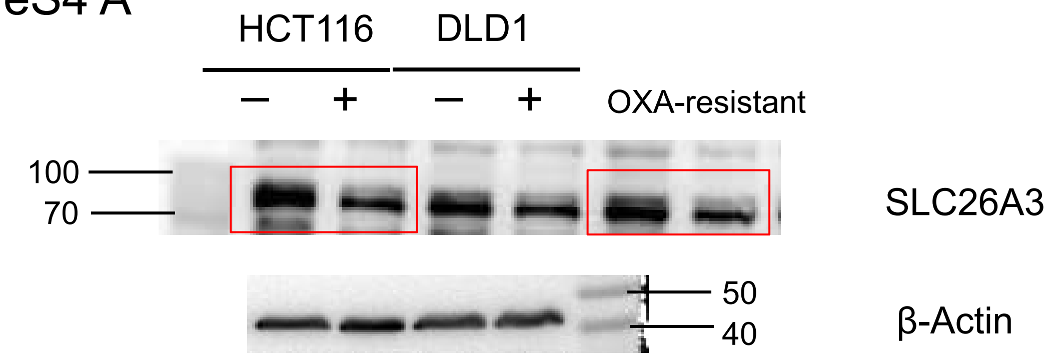

Figure6 B

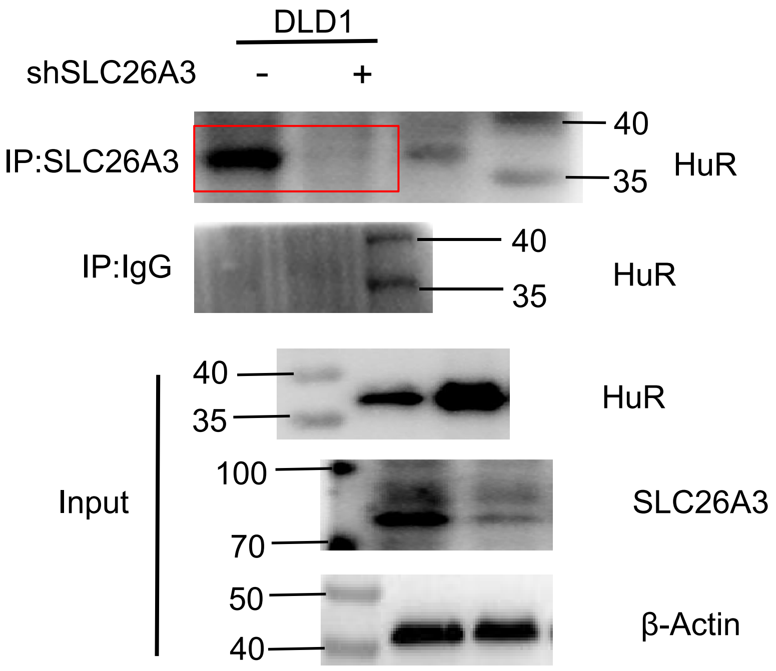

Figure6 J

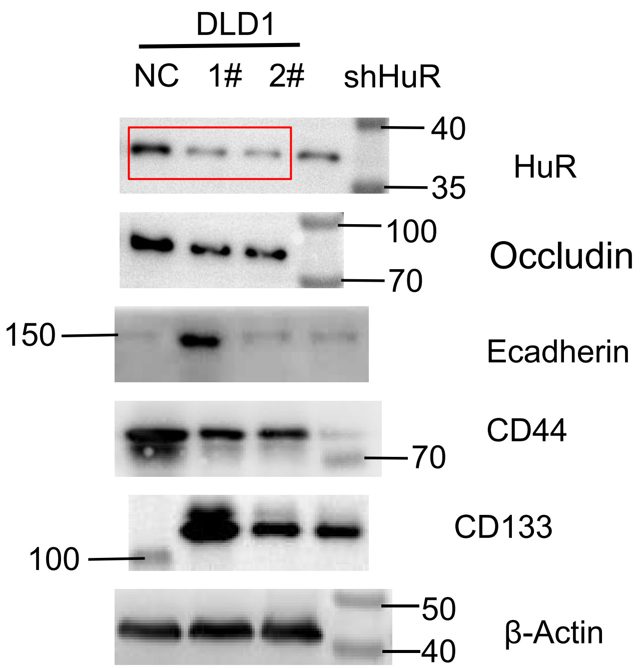

Figure6 L

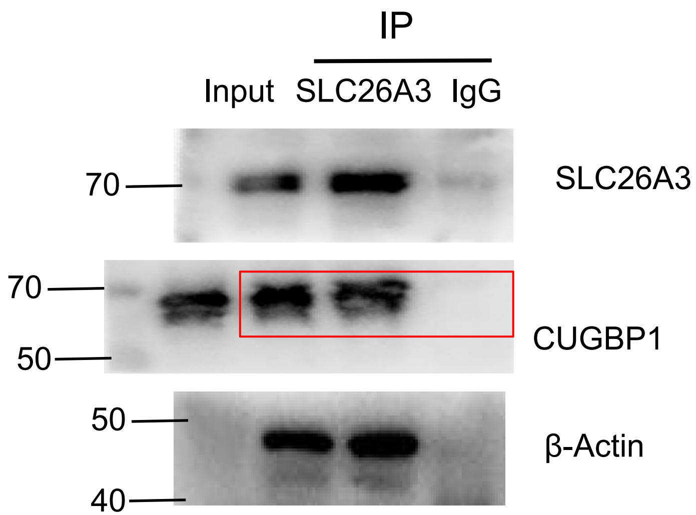

FigureS6 C

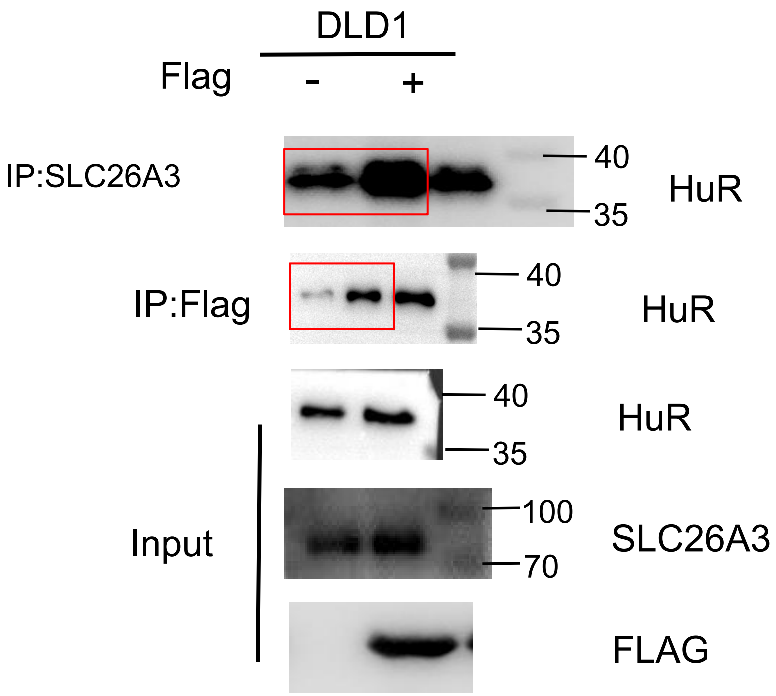

FigureS6 H

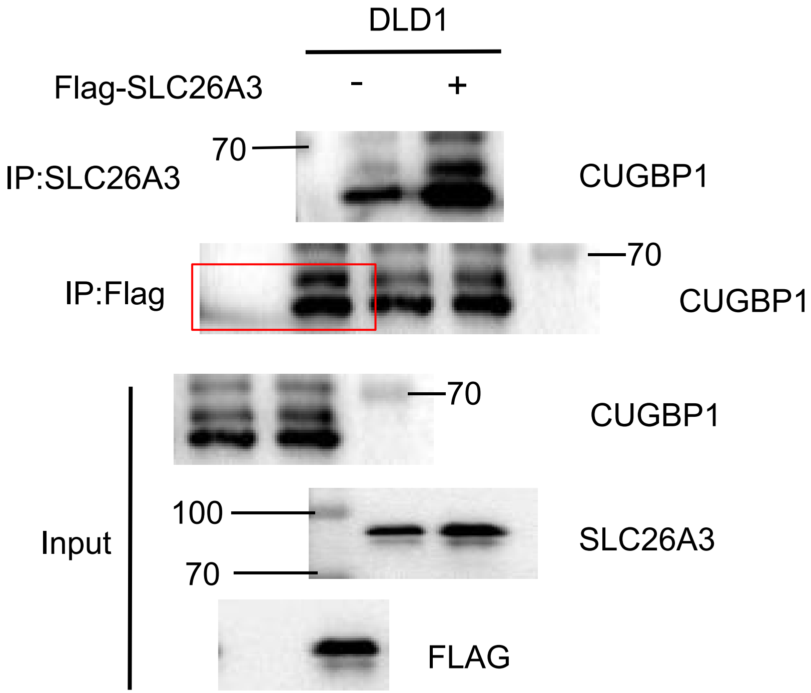

FigureS6 I

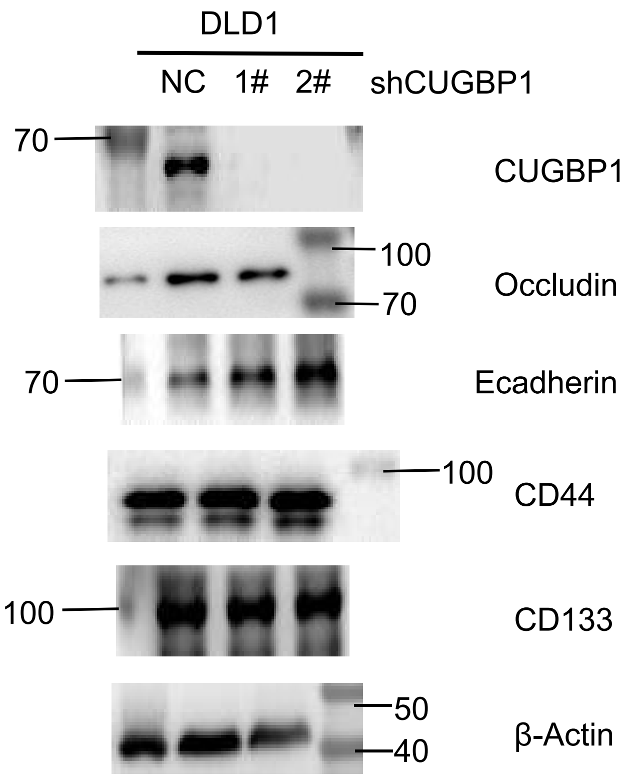

Supplement: Supplementary file 3 — Original data [file 41419_2026_8422_MOESM3_ESM.pdf]
